# A Rationally Engineered Spleen‐Tropic One‐Component Lipid‐mRNA Complex (OncoLRC) for Cancer Vaccines

**DOI:** 10.1002/advs.202512535

**Published:** 2025-11-21

**Authors:** Qimeng Yin, Chenchen Zhang, Jiahao Li, Kun Huang, Min Qiu

**Affiliations:** ^1^ Human Phenome Institute Fudan University Shanghai 201203 China; ^2^ Center for mRNA Translational Research Fudan University Shanghai 200438 China; ^3^ Zhangjiang mRNA Innovation and Translation Center Shanghai 201203 China

**Keywords:** lipid nanoparticles, mRNA cancer vaccine, one‐component lipid‐mRNA complex, spleen‐targeted delivery

## Abstract

mRNA vaccines offer great potential for cancer immunotherapy, yet efficient delivery of antigen‐encoding mRNA to antigen‐presenting cells (APCs) in lymphoid organs remains a significant challenge. Here, OncoLRC is introduced, a rationally engineered, spleen‐tropic, one‐component lipid‐mRNA complex that selectively delivers mRNA to splenic APCs following systemic administration. Through a systemic screening and optimization process, a dimethylamino (DMA)‐lipidoid‐based OncoLRC formulation that achieves nearly exclusive spleen‐targeting mRNA delivery, outperforming its conventional four‐component lipid nanoparticle (LNP) formulation counterpart has been developed. Notably, OncoLRC requires a reduced lipid‐to‐mRNA weight ratio of 1.5:1 compared to the typical 10:1 ratio in standard LNPs. OncoLRC formulated with ovalbumin (OVA) mRNA (OncoLRC*^OVA^*) promotes dendritic cell (DC) maturation and activation, eliciting robust antigen‐specific immune responses. Mechanistic studies suggest that splenic delivery is mediated primarily via macropinocytosis. Moreover, OncoLRC*^OVA^* enhances the secretion of endogenous cytokines such as IL‐12, further stimulating T cell activation and cytotoxic activity. In the B16F10‐OVA cold tumor model, OncoLRC*^OVA^* demonstrates strong prophylactic antitumor efficacy and exhibits a profound synergistic effect when combined with immune checkpoint blockade therapy, leading to significant tumor growth inhibition. Collectively, our findings establish OncoLRC as a simple yet effective APC‐targeted mRNA delivery platform, highlighting its potential as a next‐generation mRNA cancer vaccine system.

## Graphical abstract

OncoLRC, a one‐component lipid‐mRNA complex, enables efficient spleen‐targeted delivery at an exceptionally low lipid‐to‐mRNA mass ratio (1.5:1), robustly activates immune responses, inhibits tumor growth, and synergizes with checkpoint blockade, presenting a next‐generation platform for mRNA vaccines.

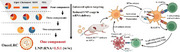

## 1 Introduction

The COVID‐19 pandemic has highlighted the transformative potential of mRNA technology, exemplified by the clinical success of Pfizer‐BioNTech and Moderna's mRNA vaccines.^[^ ^1^, ^2^, ^3^ ^]^ These vaccines have shown unprecedented clinical efficacy in preventing COVID‐19, primarily due to their ability to rapidly induce robust immune responses.^[^ ^1^, ^4^ ^]^ This success has catalyzed the development of mRNA‐based cancer vaccines as a promising strategy for eliciting potent and specific anti‐tumor immune responses.^[^ ^5^, ^6^ ^]^ mRNA vaccines can engage both innate and adaptive immune systems, providing comprehensive anti‐tumor effects.^[^ ^7^ ^]^ The primary mechanism underlies the translation of the encoded specific tumor‐associated antigens into proteins by host cells and subsequently presented on the cell surface via major histocompatibility complex (MHC) molecules.^[^ ^8^, ^9^ ^]^ In this process, antigen‐presenting cells (APCs) such as dendritic cells (DCs) and macrophages, uptake and express antigens and present them to CD4^+^ T cells via MHC‐II or to CD8^+^ T cells via MHC‐I. Additionally, they produce endogenous cytokines, such as interleukin‐12 (IL‐12), which play a pivotal role in T cell activation.^[^ ^10^, ^11^ ^]^ Activated CD4^+^ and CD8^+^ T cells release interferon‐γ (IFN‐γ) to amplify immune responses, with CD8^+^ T cells exerting potent cytotoxic effects on cancer cells through MHC‐I peptide/TCR interactions.^[^ ^12^, ^13^ ^]^ Furthermore, activated helper T cells secrete cytokines that further enhance immune responses, boosting cytotoxic T lymphocyte activity.^[^ ^14^ ^]^ Therefore, the key to the success of mRNA cancer vaccine development is to effectively transport antigen‐encoding mRNA to APCs to evoke robust antigen‐specific cellular and humoral immunity.^[^ ^15^ ^]^

Conventional prophylactic vaccines, including the SARS‐CoV‐2 mRNA vaccine, are usually injected intramuscularly to transfect the local or recruited APCs at the injection site. These transfected APCs were then transported to the draining lymph nodes to initiate immune responses.^[^ ^16^, ^17^ ^]^ However, this approach often induces slow immune activation that may not be sufficient when encountering rapidly proliferating tumors.^[^ ^18^, ^19^ ^]^ Recent studies suggested that the direct delivery of mRNA vaccines to the spleen could be an attractive approach to elicit a rapid and robust inflammatory response and T cell‐mediated immunity for cancer immunotherapy.^[^ ^20^ ^]^ As the largest peripheral lymphoid organ, the spleen has a high density of APCs close to B and T lymphocytes that provides the ideal microenvironment for efficient priming and amplification of T cell responses, making the spleen an ideal target organ for vaccinations.^[^ ^21^, ^22^ ^]^ However, the systemic delivery of antigenic mRNA to the splenic APCs remains challenging.

Lipid nanoparticles (LNPs) represent the most advanced delivery system for mRNA vaccines.^[^ ^23^ ^]^ Despite their success, traditional LNPs face a significant limitation: their tendency to accumulate in the liver following systemic administration.^[^ ^24^, ^25^, ^26^ ^]^ Significant efforts have been made to develop spleen‐selective mRNA LNP delivery systems. One approach involves incorporating anionic lipids into the LNP formulation,^[^ ^27^, ^28^, ^29^ ^]^ while another strategy is to design new ionizable lipids with intrinsic spleen‐tropism.^[^ ^28^, ^29^ ^]^ However, classical LNPs are four‐component formulations composed of ionizable lipids, cholesterol, phospholipids, and PEG‐lipids, and are often considered complex formulations.^[^ ^23^, ^30^ ^]^ Recent studies demonstrated the possibility of using the one‐component LNP system for systemic mRNA delivery to splenic T cells^[^ ^31^ ^]^ and fibroblastic reticular cells in the spleen.^[^ ^32^ ^]^

In this study, we introduce a one‐component lipid‐RNA complex system, termed OncoLRC, designed to overcome the complexity of traditional four‐component LNP formulations and enable efficient systemic mRNA delivery to splenic APCs for cancer vaccines. We first synthesized a series of DMA‐lipidoids and identified a lead candidate, H2T7, which exhibited a relatively high spleen‐to‐liver mRNA expression ratio in vivo. Through a systematic four‐step formulation screening and optimization process, we developed an optimized OncoLRC formulation that achieved nearly exclusive spleen‐targeting mRNA delivery, outperforming its conventional four‐component LNP counterpart. Notably, OncoLRC features a reduced ionizable lipid‐to‐mRNA weight ratio of 1.5:1, in contrast to the commonly used lipid‐to‐mRNA weight ratio of 10:1 in most LNPs. Using ovalbumin (OVA) mRNA as a model antigen, the OVA mRNA‐encapsulated OncoLRC vaccine (OncoLRC*^OVA^*) efficiently induced APC maturation and activation, triggering a rapid and robust antigen‐specific immune response in vivo. Additionally, OncoLRC*^OVA^* also promotes the secretion of endogenous cytokines such as IL‐12, further enhancing T cell activation and cytotoxic activity. The OncoLRC*^OVA^* vaccine demonstrates potent tumor growth inhibition, both as monotherapy and in combination with immune checkpoint blockade therapy, in the B16F10‐OVA cold tumor model. Our findings establish OncoLRC as a simple yet effective APC‐specific mRNA delivery system, highlighting its potential as a next‐generation mRNA vaccine platform for cancer immunotherapy.

## 2 Results

### 2.1 Screening and Optimization of One‐Component Lipid‐mRNA Complex (OncoLRC) for Spleen‐Tropic mRNA Delivery

**Figure 1:**
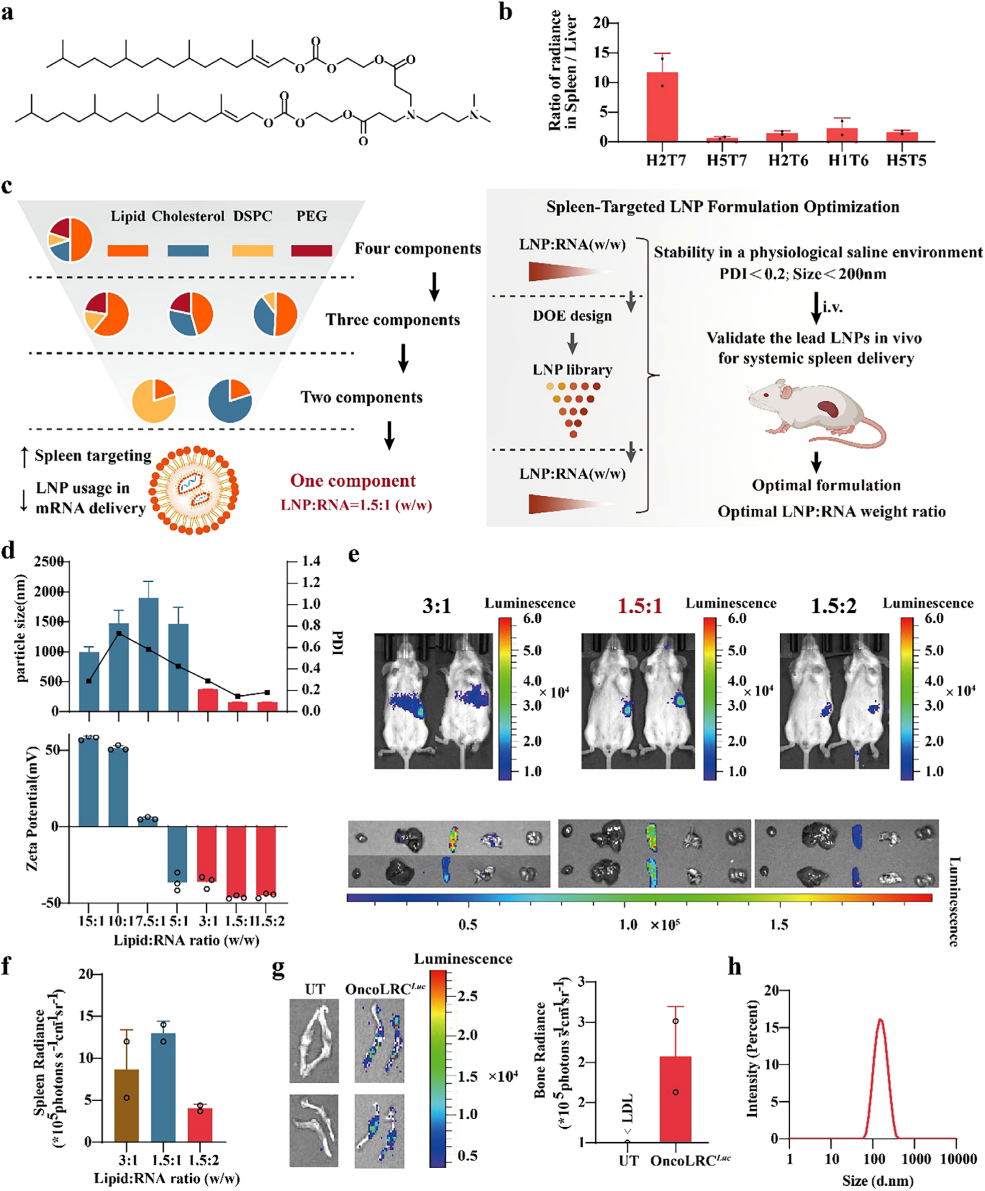
Screening and optimization of one‐component lipid‐mRNA complex (OncoLRC) for splenic antigen‐presenting cells‐selective mRNA delivery. a) Chemical structure of H2T7. b) The spleen‐to‐liver mRNA expression ratio of DMA‐lipidoids. c) Schematic of the formulation screening and optimization process for the identification of OncoLRC for spleen‐tropic mRNA delivery. d) Particle size, polydispersity index (top), and zeta potential (bottom) of OncoLRC*^Luc^* at various mass ratios (*n* = 3). e) Bioluminescence imaging of BALB/c mice 6 h after intravenous (*i.v*.) injection of OncoLRC*^Luc^* at various mass ratios. f) Quantification of bioluminescence intensity in spleen 6 h post‐treatment with OncoLRC*^Luc^*. g) Bioluminescence imaging of the femur, tibia, and knee joint in BALB/c mice after *i.v*. injection of OncoLRC*^Luc^*, with quantification of bioluminescence intensity in bone 6 h after treatment. LDL, lower detection limit. h) Size distribution of the OncoLRC*^Luc^*. Data are presented as mean ± SD.

Spleen‐specific mRNA delivery represents a promising strategy for cancer vaccines.^[^ ^33^ ^]^ With the aim to develop a one‐component spleen‐tropic mRNA lipid nanoparticle (LNP) delivery system, we first synthesized a series of lipidoids with dimethylamino (DMA) group as the ionizable head (Figure S1, Supporting Information). DMA and its analogs are key structural motifs in lipid chemistry; for example, the FDA‐approved ionizable lipid MC‐3 contains a DMA head,^[^ ^34^, ^35^ ^]^ and we recently demonstrated that rationally designed DMA‐based lipidoids enable highly efficient, spleen‐selective mRNA delivery.^[^ ^36^ ^]^ Leveraging this design, we first formulated these lipidoids with cholesterol, Distearoyl‐sn‐glycero‐3‐phosphorylcholine (DSPC), and 1,2‐dimyristoyl‐rac‐glycero‐3‐methoxypolyethylene glycol‐2000 (DMG‐PEG2k) at a molar ratio of 50:38.5:10:1.5 (lipidoid/cholesterol/DSPC/DMG‐PEG2k) to produce LNPs for initial screening. Using firefly luciferase mRNA (fLuc mRNA) as a model, we then intravenously (*i.v*.) injected the fLuc mRNA encapsulated LNPs into Balb/c mice and measured the mRNA expression using an in vivo imaging system (IVIS). As shown in Figure S1 (Supporting Information), mRNA was mainly expressed in the liver and spleen in all LNPs‐treated mice. Among them, H2T7 LNP (structure shown in **Figure** **1**a) exhibited the highest spleen‐to‐liver mRNA expression ratio (Figure 1b) and was selected as the lead candidate for further optimization. Previous studies have suggested that the delivery efficacy and the organ tropism of LNPs can be improved through formulation optimization.^[^ ^37^, ^38^, ^39^ ^]^ To achieve our goal of developing a one‐component LNP system, we designed a systemic optimization workflow, as outlined in Figure 1c. It is shown that the surface charge of LNP‐mRNA complexes plays a critical role in their targeting ability and transfection efficiency.^[^ ^27^, ^40^, ^41^ ^]^ Specifically, nanoparticles with an excess negative charge have been shown to possess favorable pharmacokinetic properties and selectively express antigens in splenic cell populations.^[^ ^27^ ^]^ Based on these insights, we first adjusted the lipid/mRNA ratio using a standard formulation composed of H2T7, cholesterol, DSPC, and PEG2000 (50:38.5:10:1.5) to form colloidally stable nanoparticles. These nanoparticles exhibited reproducible sizes (190–300 nm) and consistent surface charges (Figure S2a, Supporting Information). However, LNP‐mRNA with a slightly positive charge at a 5:1 mass ratio was unstable and immediately formed large aggregates upon preparation, consistent with previous reports.^[^ ^41^ ^]^ Although reducing the proportion of active lipid somehow improved spleen targeting, it did not eliminate fLuc expression in the liver (Figure S2b,c, Supporting Information). Motivated by emerging evidence that conventional LNP components such as cholesterol, phospholipids, and PEG may not be essential for RNA delivery and could contribute to hepatic accumulation,^[^ ^42^, ^43^, ^44^, ^45^, ^46^, ^47^ ^]^ we next investigated three‐component formulations. Specifically, we individually eliminating cholesterol, DSPC, or PEG2000 from the standard formulation. Using a design of experiments (DOE) approach, we generated nearly 40 variant formulations by adjusting the molar ratios of the remaining components and rigorously screened for optimal physicochemical characteristics (size <200 nm, PDI <0.2) and improved splenic targeting in vivo. As shown in Figure S3a–d (Supporting Information), formulations lacking either cholesterols or PEG exhibited poor colloid stability. In contrast, formulations without DSPC were more colloidally stable but displayed significantly reduced splenic targeting compared to the standard four‐component formulation at a 10:1 lipid to mRNA mass ratio (Figure S3e–h, Supporting Information). Given these results, we further explored two‐component formulations by varying the lipid/cholesterol and lipid/DSPC molar ratios ranging from 5:1 to 1:5. However, these formulations also exhibited poor colloid stability (Figure S4a,b, Supporting Information). Moreover, due to concerns about the potential immunogenicity of PEGylated nanoparticles, specifically the risk of inducing anti‐PEG antibodies such as IgG and IGM that can lead to accelerated blood clearance (ABC), reduced therapeutic efficacy, and potential allergic reactions,^[^ ^48^, ^49^, ^50^ ^]^ we excluded PEG from our two‐component formulation studies.

Finally, we investigated the use of H2T7 active lipid as the sole component of the LNP formulation and explored how different lipid/mRNA mass ratios affected the colloidal stability and spleen targetability of the resulting LNP‐mRNA complexes. We observed that as the lipid/mRNA ratio decreased from 15/1 to 7.5/1, the size of the LNP‐mRNA complex increased from ≈500 nm to nearly 2000 nm. Further reduction of the lipid/mRNA led to a decrease in particle size, with nanoparticles exhibiting optimal physicochemical properties, size<200 nm, PDI<0.2, and a negatively charged surface, when the lipid/mRNA ratio reached 1.5/1 or 1.5/2 (Figure 1d). Interestingly, gradually reducing the active lipid content resulted in a decrease in fLuc expression in the liver, while simultaneously increasing expression in the spleen (Figure 1e). When the lipid/mRNA (w/w) ratio fell below 3:1, nearly exclusive splenic signals were detected (Figure 1e). Among these, the 1.5:1 ratio exhibited the most pronounced spleen targeting and signal intensity (Figure 1e,f), with luciferase activity also detected in the femur and tibia bone marrow (Figure 1g). Importantly, this bone marrow localization represents a potential immunological advantage rather than off‐target delivery. As the central site of hematopoiesis and a long‐term niche for memory T and B cells, bone marrow targeting may broaden the antigen‐responsive repertoire and support durable immune memory.^[^ ^51^, ^52^ ^]^ Thus, the dual engagement of both spleen and bone marrow, corresponding to immediate immune priming and long‐term immunity maintenance, positions our system as a promising platform for eliciting both potent and sustained antitumor responses.

Based on these findings, we selected one component lipid‐mRNA complex (OncoLRC) for further experiments in cancer vaccines. Compared to traditional four‐component formulations, OncoLRC offers several advantages, including a simplified composition and a significantly reduced lipid‐to‐mRNA mass ratio (1.5:1). The reduced lipid content minimizes the potential toxicity risks associated with lipid components in mRNA vaccine delivery. As shown in Figure 1h and Table S1 (Supporting Information), OncoLRC has a size of 158 nm, a narrow PDI, and an mRNA encapsulation efficiency of ≈85%. Electron microscopy revealed that OncoLRC nanoparticles possess a spherical morphology with an electron‐dense core (Figure S5, Supporting Information). Notably, OncoLRC exhibited excellent storage stability at 4 °C, maintaining both its physicochemical properties and in vivo mRNA delivery efficacy for at least 10 days (Figure S6a–c, Supporting Information). More importantly, repeated administration of OncoLRC encapsulating fLuc mRNA (0.25 mg/kg^−1^, five doses) did not alter in vivo tropism or reduce splenic luciferase signals (Figure S6d,e, Supporting Information), indicating that the delivery efficiency of the OncoLRC was preserved over multiple administrations without evidence of accelerated clearance or loss of targeting ability. To further evaluate its versatility, OncoLRC was used to encapsulate and deliver more complex RNA constructs, including circular RNA (circRNA) encoding luciferase and a larger, therapeutically relevant mRNA encoding CAR‐hHER2‐fLuc (3463 bp). As shown in Figure S7 (Supporting Information), OncoLRC enables spleen‐tropic delivery of both constructs. Collectively, these results demonstrate that OncoLRC is a highly versatile and promising platform for mRNA‐based therapeutics.

### 2.2 OncoLRC Successfully Delivered mRNA to Splenic Antigen‐Presenting Cells (APCs)

**Figure 2:**
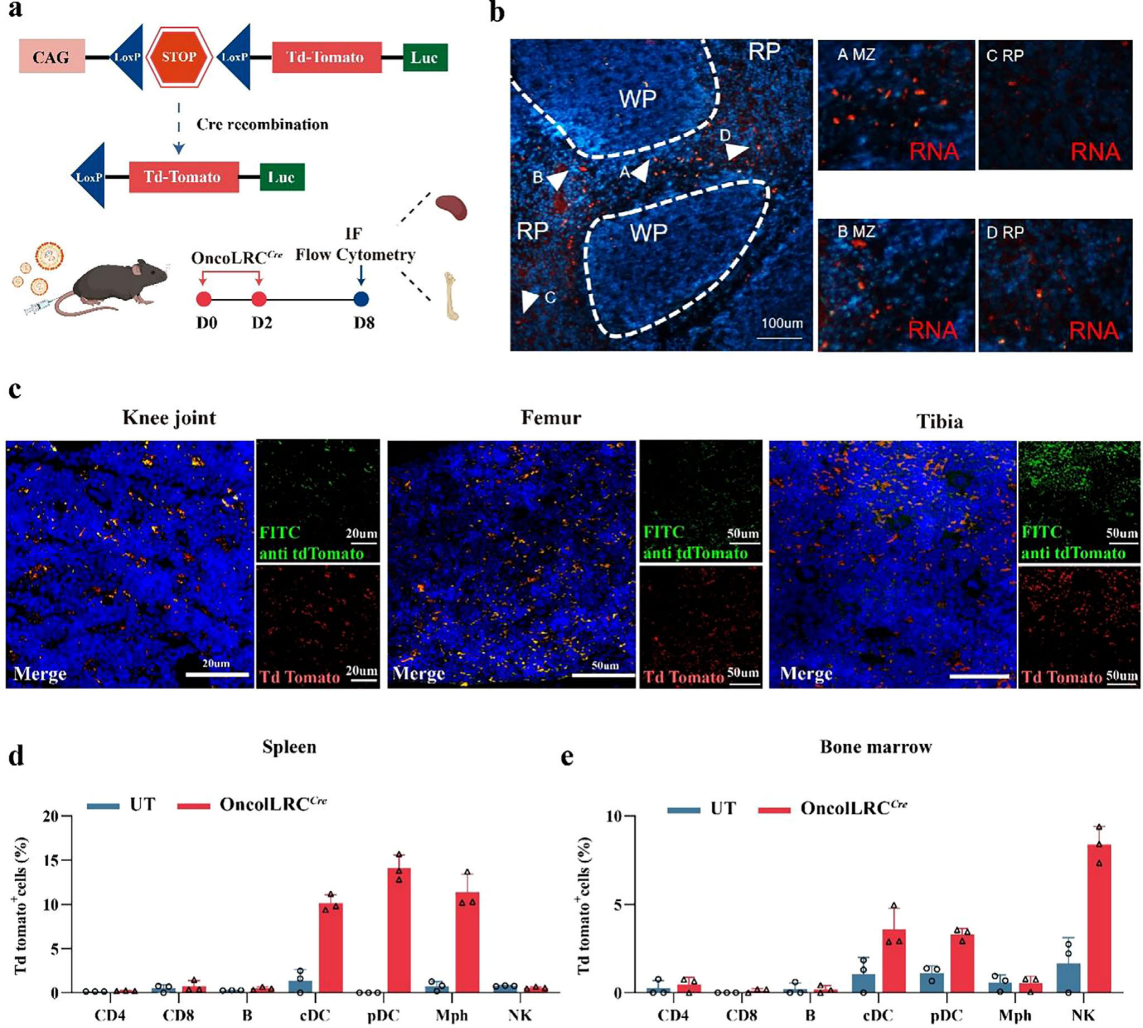
OncoLRC successfully delivered mRNA to antigen‐presenting cells (APCs) in the spleen. a) Schematic illustration of Cre recombinase via LNP‐mediated Cre mRNA to delete the stop cassette in R26‐CAG‐LSL‐Luc‐tdTomato Cre‐reporter mouse to activate tdTomato expression. Representative images of tdTomato‐positive cells in the spleen b) and bone marrow c) of Cre‐reporter mice after *i.v*. injection of OncoLRC*^Cre^*. MZ, marginal zone; RP, red pulp; WP, white pulp. Percentage of tdTomato‐positive cells among different immune cell populations in the spleen d) and bone marrow e). Data are presented as mean ± SD.

To identify the specific cell types targeted by OncoLRC, we administered Cre mRNA‐encapsulated OncoLRC to genetically engineered Cre‐reporter mice carrying a LoxP‐flanked STOP cassette regulating the expression of tdTomato. Five days post‐injection, the mice were euthanized, and spleen and bone marrow were collected for immunofluorescence staining and flow cytometry analyses (**Figure** **2**a). Immunofluorescence staining of the spleen showed tdTomato expression in CD11c^+^ conventional dendritic cells (cDCs) located in the marginal zone, as well as in plasmacytoid dendritic cells (pDCs) and macrophages (Figure 2b). Similarly, immunofluorescence analysis of bone marrow also revealed tdTomato‐positive cells in the femur, tibia, and knee joint (Figure 2c; Figure S8, Supporting Information). Flow cytometry was further performed to quantify tdTomato expression across key immune cell subpopulations in both tissues. The corresponding gating strategy and representative flow cytometry plots are shown in Figures S9 and S10 (Supporting Information). The results confirmed robust transfection of DCs in both spleen and bone marrow (Figure 2d,e). In addition, tdTomato expression was observed in a subset of splenic macrophages and a substantial proportion of bone marrow natural killer (NK) cells.

### 2.3 Mechanistic Insights into Cellular Internalization of OncoLRC

**Figure 3:**
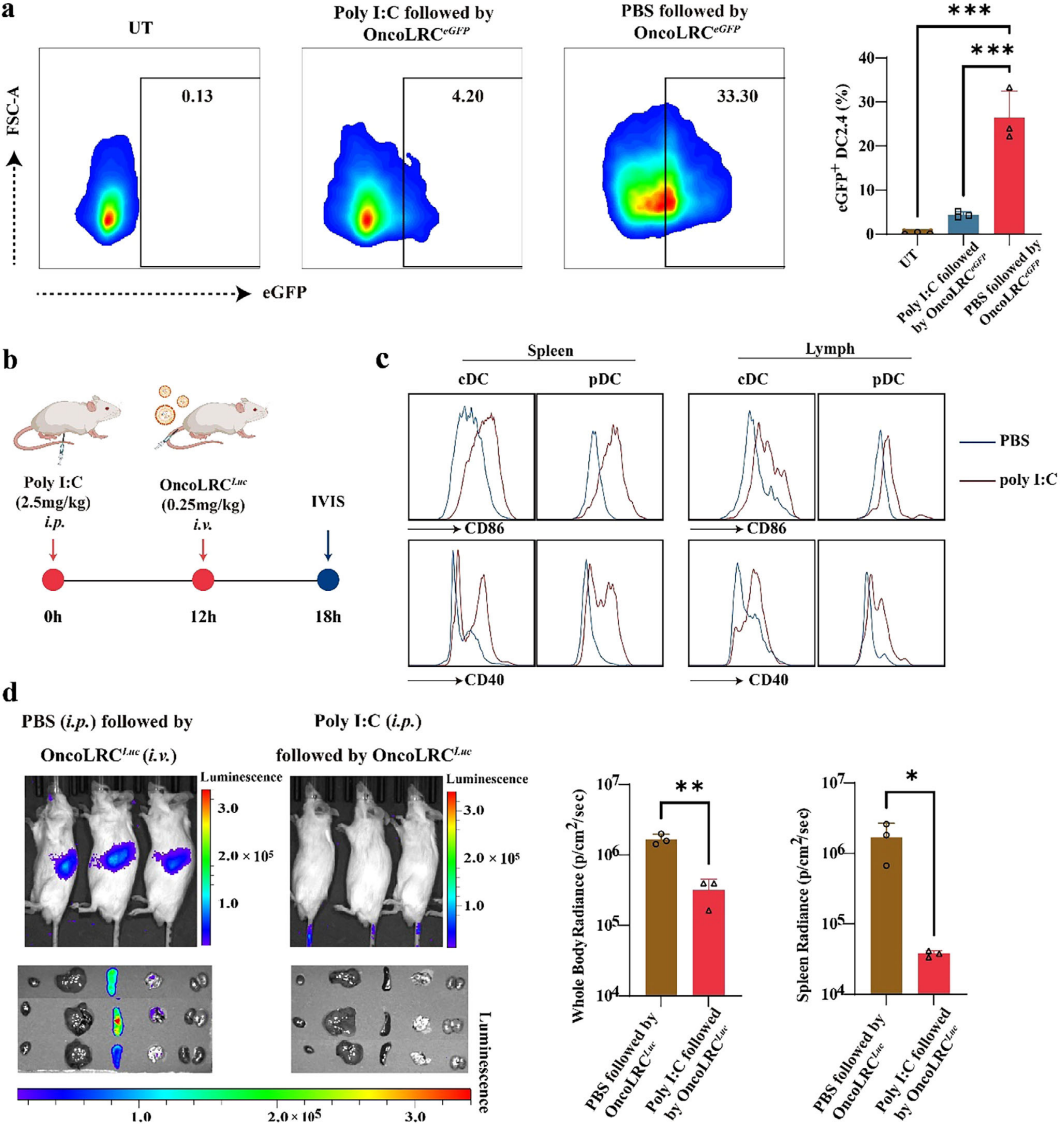
Mechanistic insights into cellular internalization of OncoLRC. a) Quantification of eGFP‐positive cells following transfection with OncoLRC*^eGFP^*, with or without poly I: C pretreatment. b) Schematic of the in vivo experimental design: Intraperitoneal poly(I: C) (0.25 mg kg^−1^) pretreatment induces DC maturation, followed by intravenous OncoLRC*^Luc^* (0.25 mg kg^−1^) administration 12 h later to assess macropinocytosis‐dependent cellular uptake. c) Analysis of activation markers in splenic and lymph node immune cell subsets 12 h after poly I: C injection. d) Representative bioluminescence images of BALB/c mice with or without poly I: C pretreatment following *i.v*. injection of OncoLRC*^Luc^*, along with quantification of whole‐body and splenic bioluminescence intensity at 6 h after OncoLRC*^Luc^* treatment (*n* = 3). Significance was determined using one‐way ANOVA and Tukey's multiple comparisons test (a), and Student's *t*‐test (d). Data are presented as mean ± SD. *p < 0.05, **p < 0.01, ***p < 0.001, ****p < 0.0001.

To elucidate the cellular uptake mechanism of OncoLRC, we systematically investigated its internalization pathways both in vitro and in vivo. Previous studies have shown that immature dendritic cells (DCs) constitutively internalize naked RNA through macropinocytosis, a pathway that is markedly suppressed upon DC maturation, whereas phagocytic and receptor‐mediated endocytic activities remain largely unaffected.^[^ ^53^, ^54^ ^]^ In line with these findings, uptake of OncoLRC*^eGFP^* by polyinosinic: polycytidylic acid (poly(I: C))‐matured DC2.4 cells was significantly reduced compared to that in untreated, immature DCs (**Figure** **3**a). To validate these observations in vivo, we pretreated female Balb/C mice with an intraperitoneal injection of poly(I: C) (0.25 mg kg^−1^) or PBS as control 12 h before intravenous injection of OncoLRC*^Luc^* (0.25 mg kg^−1^) (Figure 3b). In line with the previous report, poly(I: C) treatment induced maturation of DCs in the spleen and lymph nodes (Figure 3c), thereby suppressing macropinocytosis. Corroborating this mechanism, poly(I: C) pretreatment resulted in a dramatic reduction (>98%) in splenic luciferase signal intensity relative to the PBS control group (Figure 3d). Collectively, these results indicate that macropinocytosis serves as the primary pathway for OncoLRC internalization in splenic antigen‐presenting cells.

### 2.4 OncoLRC Vaccine Induces Robust Activation of DCs In Vivo

**Figure 4:**
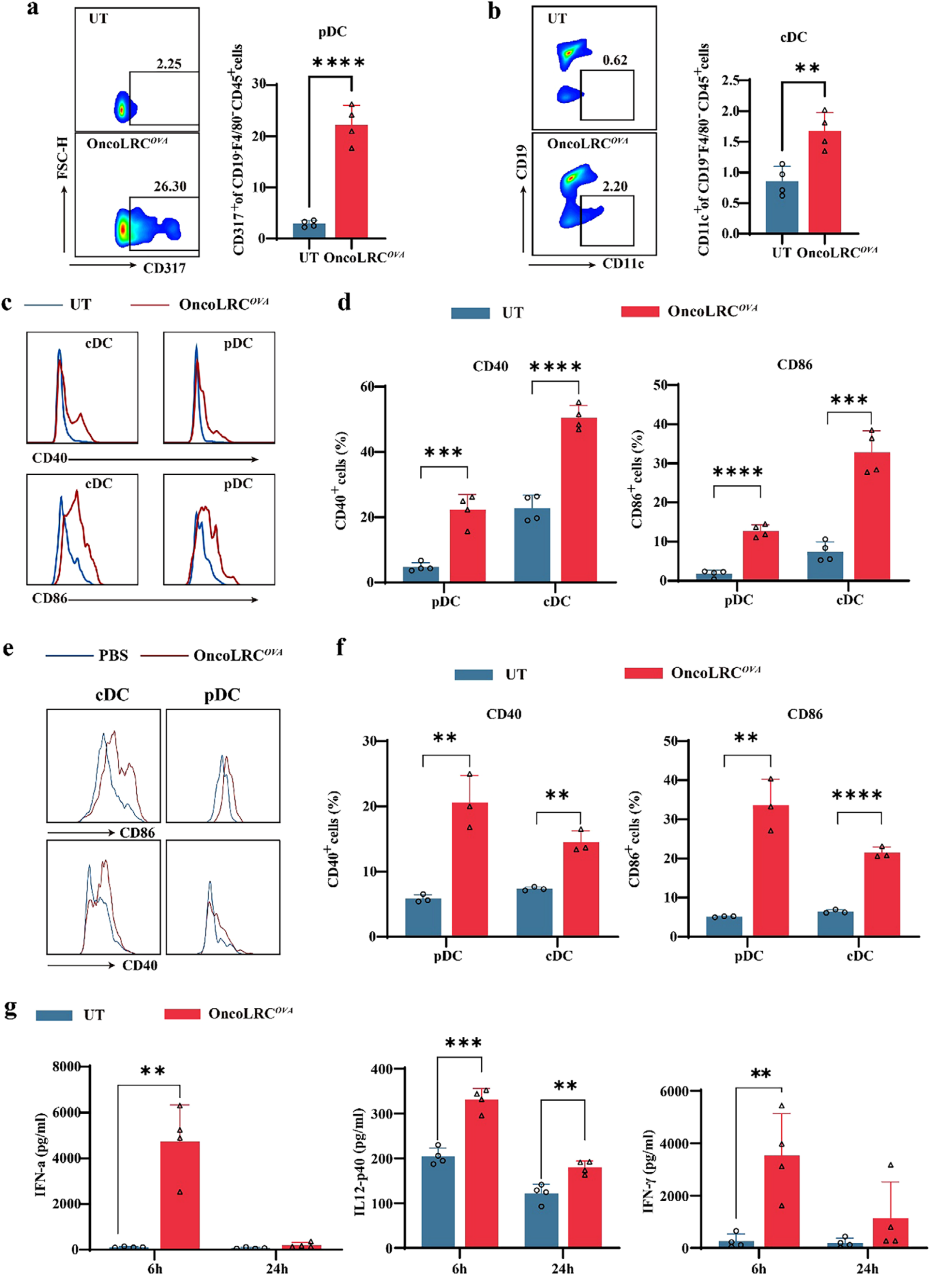
OncoLRC vaccine induces activation of DCs. Quantification of pDC a) and cDC b) in the spleen 24 h post‐vaccination with OncoLRC*^OVA^*. c) Expression of activation markers in splenic immune cell subsets 24 h post‐vaccination. d) Percentage of CD40^+^ and CD86^+^ pDCs and cDCs in the spleen 24 h post‐vaccination. e) Expression of activation markers in lymph node immune cell subsets 24 h post‐vaccination. f) Percentage of CD40^+^ and CD86^+^ pDCs and cDCs in the lymph node 24 h post‐vaccination. g) Serum levels of IFN‐α, IL‐12p40, and IFN‐γ in C57 mice at 6 and 24 h post‐vaccination. (*n* = 4). Significance was determined using Student's *t*‐test (a, b, d, f, g). Data are presented as mean ± SD. *p < 0.05, **p < 0.01, ***p < 0.001, ****p < 0.0001.

To further assess the immunostimulatory effects of OncoLRC in vivo, we administered a single intravenous (*i.v*.) injection of OncoLRC encoding ovalbumin (OVA), a well‐established model antigen for evaluating antigen‐specific immune responses. At 24 h post‐injection, we observed a significant increase in the proportion of plasmacytoid dendritic cells (pDCs) (**Figure** **4**a) and conventional dendritic cells (cDCs) in the spleen (Figure 4b). pDCs are critical mediators of antiviral immunity, while cDCs are central to antigen presentation and T cell activation.^[^ ^55^, ^56^ ^]^ In addition to their increased abundance, both pDCs and cDCs exhibited upregulated expression of activation markers CD40 and CD86 (Figure 4c,d), indicative of dendritic cell maturation and their enhanced capacity to initiate T cell responses. In addition, both pDCs and cDCs in the lymph nodes also displayed significantly increased expression of the activation markers CD40 and CD86 (Figure 4e,f). Concurrently, serum levels of key cytokines, including IFN‐α, IFN‐γ, and IL‐12p40, peaked at 6 h, further supporting the robust systemic immune activation (Figure 4g). IFN‐α contributes to antiviral defense and immune cell stimulation^[^ ^57^ ^]^ while IFN‐γ and IL‐12p40 are essential for promoting T cell‐mediated immune responses.^[^ ^58^ ^]^

We further compared the in vivo immunogenicity of OncoLRC with the well‐established cationic lipid‐based splenic DC‐targeting LPX platform developed by BioNTech.^[^ ^41^ ^]^ Consistent with previous reports, LPX had a particle size of ≈231.8 nm (Table S2, Supporting Information) and efficiently targeted the spleen after systemic administration (Figure S11a, Supporting Information). Following intravenous administration of OncoLRC*^OVA^* and LPX*^OVA^* (1 mg kg^−1^), serum levels of IFN‐α, IFN‐γ, and IL‐12p40 peaked at 6 h and returned to baseline by 24 h, with no significant differences between the two groups (Figure S11b, Supporting Information). Comparable DCs activation in the spleen for both vaccines was observed at 24 h post‐administration (Figure S11c, Supporting Information). However, OncoLRC*^OVA^* induced a higher proportion of CD40⁺ pDCs in the lymph nodes compared to LPX*^OVA^* (Figure S11d, Supporting Information).

Collectively, these results demonstrate that OncoLRC vaccination triggers robust activation of DCs in both the spleen and lymph nodes, along with a transient systemic cytokine increase. Compared with LPX, OncoLRC shows enhanced ability to activate lymph node DCs, suggesting improved capacity to initiate adaptive immune responses.

### 2.5 OncoLRC*^OVA^* Vaccine Elicits a Robust Cytotoxic CD8^+^ T Cell Response and Protective Effects in B16F10‐OVA Tumor Model

**Figure 5:**
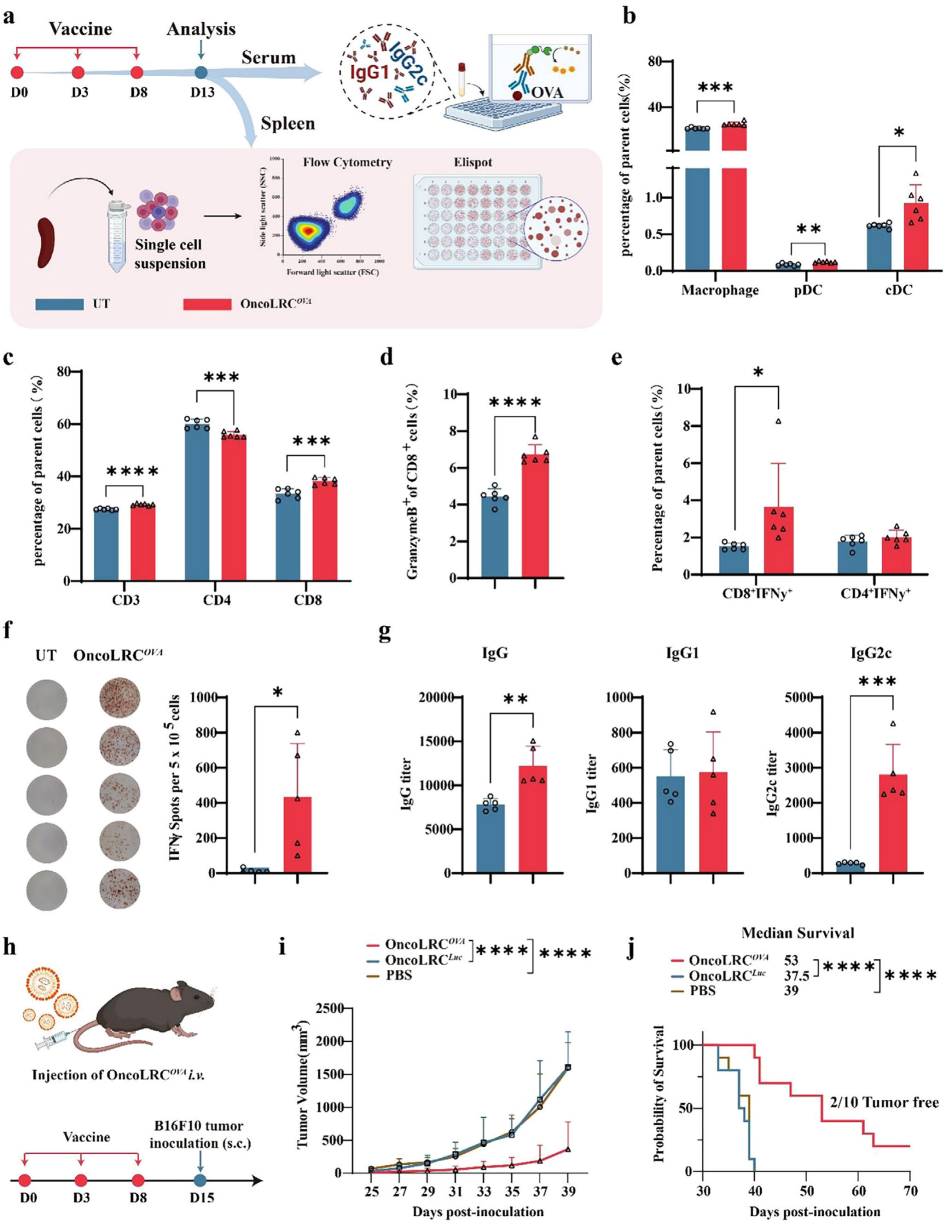
OncoLRC*^OVA^* vaccine elicits a robust T cell response and provides prophylactic effect against B16F10‐OVA tumor. a) Schematic diagram of the vaccination timeline and experimental workflow for analyzing spleen and serum samples from C57BL/6 mice 5 days after vaccination. Changes in the proportion of antigen‐presenting cells (APCs) b), T cells c), Granzyme B^+^ T cells d), and IFN‐γ^+^ T cells e) in the spleen after vaccination (*n* = 6). f) Representative ELISpot images and quantification of IFN‐γ‐secreting T cells within splenocytes of vaccinated mice (*n* = 5). g) OVA‐specific antibody titers in mice vaccinated with OncoLRC*^OVA^* (*n* = 5). h) Schematic of the experimental design for prophylactic effect of OncoLRC*^OVA^* vaccine in B16F10‐OVA tumor model. i) Tumor volume changes of mice treated with different formulations (*n* = 10). j) Kaplan‐Meier survival curves of mice in PBS, OncoLRC*^Luc^*, and OncoLRC*^OVA^* treatment groups (*n* = 10). Significance was determined using Student's *t*‐test (b–g), two‐way ANOVA and Dunnett's multiple comparisons test (i), log‐rank test (j). Error bars, mean ± SD. *p < 0.05, **p < 0.01, ***p < 0.001, ****p < 0.0001.

After observing the strong immune activation after a single dose of OncoLRC*^OVA^* in vivo, we next investigated the immune responses induced by repeated vaccination. The dosing schedule and experimental workflow are shown in **Figure** **5**a. Repeated administration of OncoLRC*^OVA^* led to a notable increase in splenic immune cell populations, including macrophages (Mph), pDCs, cDCs (Figure 5b), CD4^+^ T cells, and CD8^+^ T cells (Figure 5c). Importantly, the proportion of cytotoxic T cells, such as Granzyme B^+^ CD8^+^ T cells and IFN‐γ^+^ CD8^+^ T cells, was significantly elevated following vaccination (Figure 5d,e). To further assess antigen‐specific T cell responses, we performed an IFN‐γ ELISpot assay using SIINFEKL peptide stimulation. As shown in Figure 5f, vaccinated mice exhibited a strong IFN‐γ‐secreting T cell response, while the unvaccinated control group showed minimal activity, indicating a lack of tumor antigen recognition. We also measured the serum levels of total IgG, IgG1, and IgG2c five days after the third vaccine dose using ELISA to evaluate the humoral immune response induced by OncoLRC*^OVA^*. The total IgG and IgG2c levels were significantly elevated in vaccinated mice, but IgG1 levels were comparable between vaccinated and control groups (Figure 5g). Given that IgG2c is associated with a Th1‐biased immune response,^[^ ^59^ ^]^ this result suggests that OncoLRC*^OVA^* promotes both cellular immunity and a Th1‐skewed humoral response, enhancing the potential for effective antitumor activity.

We next assessed the prophylactic potential of OncoLRC*^OVA^* using the B16F10‐OVA tumor model. Mice were immunized on days 0, 3, and 8 as illustrated in Figure 5h, and then challenged the mice with 0.5 million B16F10‐OVA tumor cells on day 15. As shown in Figure 5i and Figure S12 (Supporting Information), tumor growth was rapid in unvaccinated mice, whereas vaccinated mice showed significantly delayed tumor progression, with 1 out of 5 mice remaining tumor‐free. Moreover, OncoLRC*^OVA^* vaccination markedly prolonged survival, extending lifespan by over two weeks compared to the control group (Figure 5j). Collectively, these results demonstrate that the OncoLRC*^OVA^* vaccine induces potent cellular and humoral immune responses, offering effective protection against tumor challenge and underscoring its potential as a therapeutic cancer vaccine.

### 2.6 Therapeutic Effect of OncoLRC*^OVA^* Vaccine in B16F10‐OVA Tumor Model

**Figure 6:**
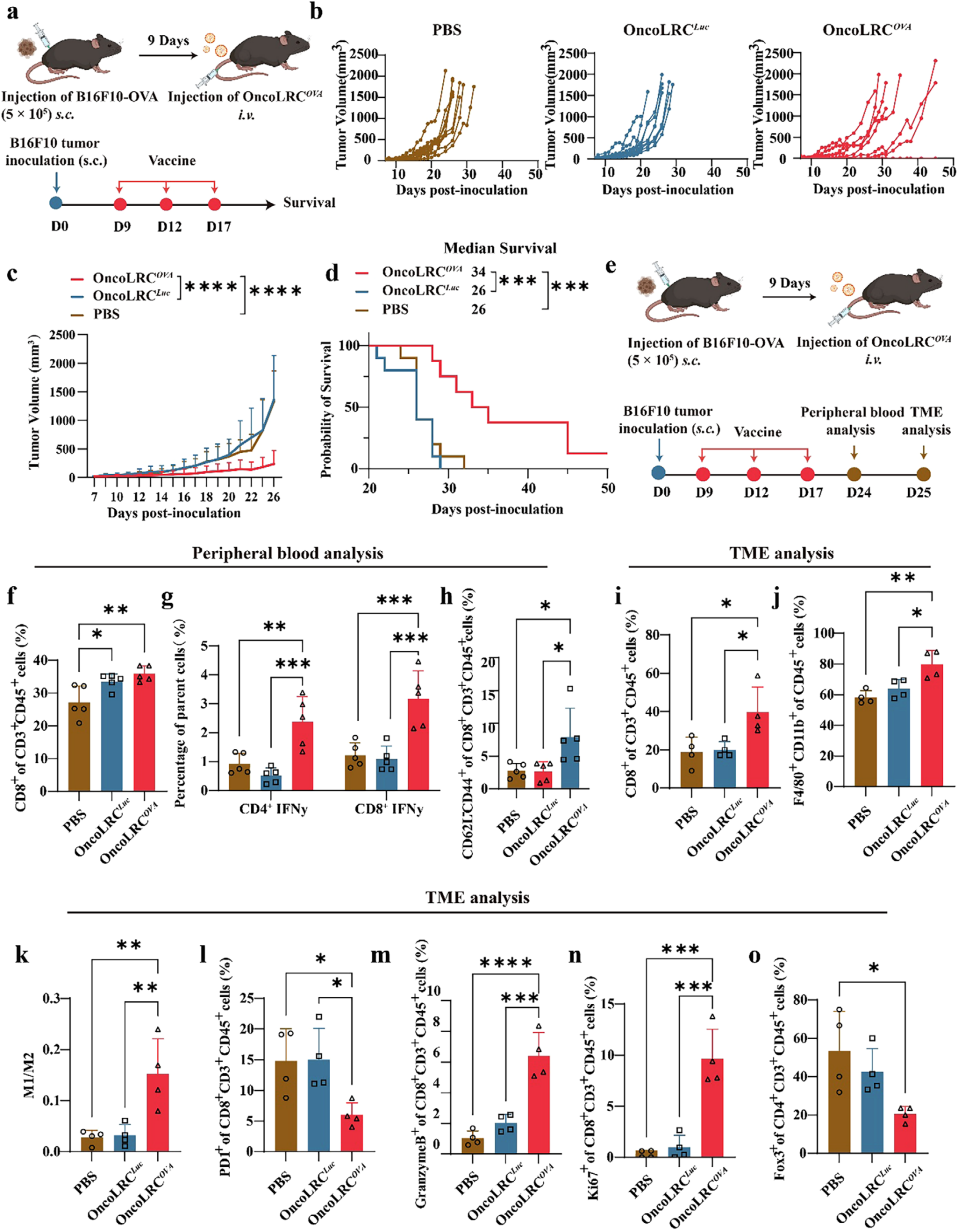
OncoLRC*^OVA^* vaccine effectively inhibits B16F10‐OVA tumor growth. a) Illustration of the treatment process for B16F10‐OVA model. b) Tumor‐growth profiles of mice in different treatment groups (*n* = 10). c) Tumor volume changes of mice treated with different formulas over time (*n* = 10). d) Kaplan‐Meier survival curves of mice in PBS, OncoLRC*^Luc^*, and OncoLRC*^OVA^* treatment groups (*n* = 10). e) Schematic of the peripheral blood and TME immune profiling. Changes in peripheral blood CD8^+^ T cell levels f), IFN‐γ^+^ T cells g), CD8^+^ TEM cells h) on day 24 (*n* = 5). Changes in CD8^+^ T cell levels i), macrophage j), M1/M2 ratio k), PD1^+^CD8^+^ cells l), Granzyme B^+^CD8^+^ cells m), Ki67^+^CD8^+^ cells n), and Treg (Foxp3^+^) cells o) within the tumor on day 25 (*n* = 4). Significance was determined using two‐way ANOVA and Dunnett's multiple comparisons test (c), log‐rank test (d), and one‐way ANOVA and Tukey's multiple comparisons test (f–o). Data are presented as mean ± SD. *p < 0.05, **p < 0.01, ***p < 0.001, ****p < 0.0001.

Encouraged by the potent prophylactic efficacy of OncoLRC*^OVA^*, we next evaluated its therapeutic potential against established tumors. In the B16F10‐OVA solid tumor model, mice were subcutaneously inoculated with 0.5 million tumor cells in the right thigh on day 0, followed by intravenous vaccination on days 9, 12, and 17, as depicted in **Figure** **6**a. As shown in Figure 6b–d, treatment with the OncoLRC*^OVA^* vaccine significantly inhibited tumor growth and extended the survival of mice. Importantly, repeated vaccine administration did not induce systemic toxicity, as indicated by stable body weight and unaltered serum levels of AST and ALT (Figure S13a,b, Supporting Information), supporting the favorable safety profile of OncoLRC*^OVA^*.

To explore vaccine‐induced immune responses, we analyzed immune cell populations under the same experimental conditions (Figure 6e). Seven days after the final vaccine dose, peripheral blood analysis revealed a significant increase in the proportion of CD8^+^ T cells (Figure 6f), as well as elevated frequencies of IFN‐γ^+^CD4^+^ T cells and IFN‐γ^+^CD8^+^ T cells (Figure 6g) following vaccination, indicating enhanced cellular immunity. Additionally, we observed a significant rise in the proportion of CD8^+^ effector memory T cells (TEM) (Figure 6h), suggesting the generation of OVA‐specific immune memory. Tumors were harvested eight days after the final vaccination to examine the tumor immune microenvironment (TME). Compared to the untreated group, vaccinated mice exhibited a marked increase in intratumoral CD8^+^ T cell infiltration (Figure 6i). Interestingly, macrophage infiltration, especially M1‐type macrophages (iNOS^+^), was significantly enhanced in vaccinated tumors (Figure 6j,k), indicating improved antigen presentation and innate immune activation.

Further phenotypic characterization of tumor‐infiltrating lymphocytes revealed a reduction in PD‐1⁺CD8⁺ T cells (Figure 6l), accompanied by an increase in Granzyme B⁺CD8⁺ T cells and proliferative Ki67⁺CD8^+^ T cells (Figure 6m,n), indicating that OncoLRC*^OVA^* vaccination alleviates tumor‐induced T cell exhaustion and enhances cytotoxic and proliferative activity. In addition, we observed a notable decrease in regulatory T cells (Tregs), identified by FoxP3 staining. In untreated tumors, ≈50% of CD4⁺ T cells were FoxP3⁺ Tregs, reflecting a highly immunosuppressive tumor microenvironment. Notably, the proportion of Tregs in the OncoLRC*^OVA^* vaccination group decreased by 60% compared to the other two treatment groups (Figure 6o), further indicating a reversal of immunosuppressive conditions. Gating strategies and representative flow cytometry plots are provided in Figures S14 and S15 (Supporting Information). Collectively, these findings indicate that OncoLRC*^OVA^* reshapes the TME by promoting effector T cell infiltration, reducing suppressive immune elements, and enhancing antitumor immunity.

### 2.7 Combination of OncoLRC and Anti‐PD‐1 Antibody Enhances Anti‐Tumor Immunity and Prolongs Survival in B16F10‐OVA Tumor Model

**Figure 7:**
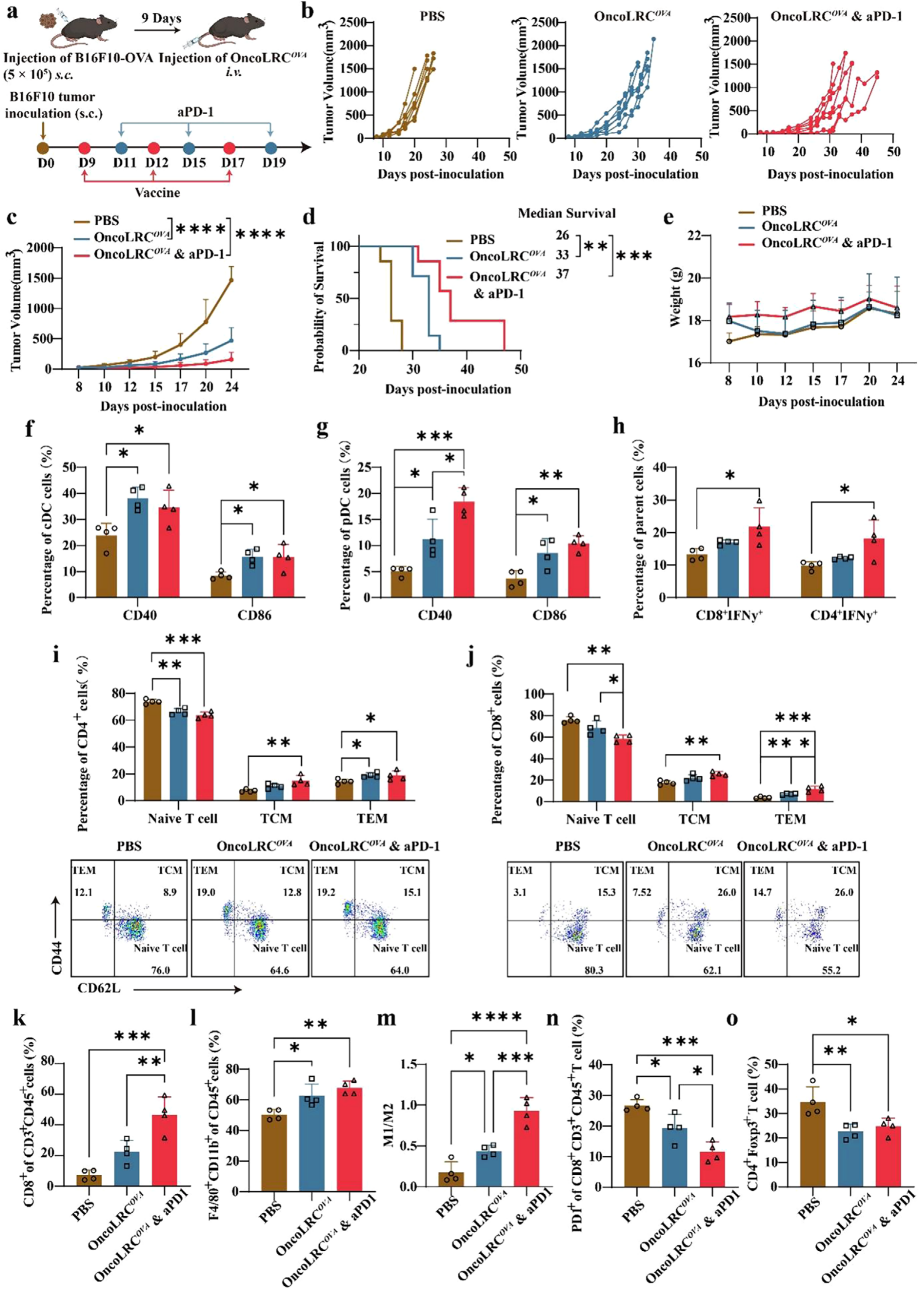
Combination of OncoLRC*^OVA^* mRNA and anti‐PD‐1 antibody synergistically enhanced antitumor efficacy in the B16F10‐OVA tumor model. a) Illustration of the combination treatment process for the B16F10‐OVA model. b) Tumor growth profiles of mice in different treatment groups (*n* = 7). c) Tumor volume of mice treated with different formulations over time (*n* = 7). d) Kaplan‐Meier survival curves of mice treated with different formulations (*n* = 7). e) Changes in mouse body weight over time (*n* = 7). Percentage of CD40^+^ and CD86^+^ cDCs f) and CD40^+^ and CD86^+^ pDCs g) in the spleen on day 24 (*n* = 4). Percentage of IFN‐y^+^ T cells h), CD4^+^ memory T cells i), and CD8^+^ memory T cells j) in the spleen on day 24 (*n* = 4). Changes in CD8^+^ T cell levels k), macrophage levels l), ratio of M1 to M2 macrophages m), the proportion of PD1^+^CD8^+^ cells n), and the proportion of Tregs (Foxp3^+^) o) within the TME on day 24 (*n* = 4). Significance was determined using two‐way ANOVA and Dunnett's multiple comparisons test (c), log‐rank test (d), and one‐way ANOVA and Tukey's multiple comparisons test (f–o). Data are presented as mean ± SD. *p < 0.05, **p < 0.01, ***p < 0.001, ****p < 0.0001.

Given the immune‐stimulating effects of OncoLRC, we next explored its therapeutic synergy with PD‐1 checkpoint blockade in the B16F10‐OVA tumor model. Mice received the same tumor inoculation and OncoLRC*^OVA^* vaccination schedule as described above. In the combination group, anti‐PD‐1 antibody (aPD‐1) was administered intraperitoneally on days 11, 15, and 19 post‐tumor inoculation (**Figure** **7**a). As shown in Figure 7b–d, we still show the OncoLRC*^OVA^* vaccine‐mediated antitumor effects, whereas the combination with aPD‐1 exhibited somehow improves the tumor growth control and the mouse survival time. Mouse body weight and serum levels of AST and ALT in all treated mice remained stable, confirming the durability of the combined therapy (Figure 7e; Figure S16, Supporting Information).

We further analyzed immune cell populations in the spleen and tumors. We found that both treatment regimens enhanced the activation of cDCs and pDCs in the spleen (Figure 7f,g). Notably, the combination therapy elicited significantly higher proportions of IFNγ^+^ CD8^+^ and CD4^+^ T cells than OncoLRC*^OVA^* monotherapy in the spleen, indicating stronger systemic T cell activation (Figure 7h). Furthermore, the combination group showed increased frequencies of CD4^+^ and CD8^+^ central and effector memory T cells (TCM and TEM), alongside a decrease in naïve T cells, suggesting the establishment of robust immune memory (Figure 7i,j). Additionally, the combination treatment induced more pronounced infiltration of T cells and APCs within the TME compared to monotherapy (Figure 7k–m). Importantly, it further reduced the proportions of immunosuppressive cells, including PD‐1^+^CD8^+^ T cells and Tregs, suggesting the reversal of immunosuppression (Figure 7n,o). Representative flow cytometry plots are shown in Figure S17 (Supporting Information). Taken together, these findings demonstrate that combination of OncoLRC and PD‐1 blockade synergistically improves the anti‐tumor immune response, resulting in prolonged survival and enhanced immune memory, highlighting the potential of this combination strategy for the treatment of “cold” solid tumors.

## 3 Discussion

Despite the promise of mRNA‐based cancer vaccines, their efficacy remains limited by challenges such as inefficient delivery to dendritic cells (DCs) within lymphoid organs^[^ ^60^ ^]^ and the liver‐centric biodistribution of conventional lipid nanoparticles (LNPs),^[^ ^24^, ^25^, ^26^, ^61^ ^]^ which restricts their applications in treating non‐hepatic malignancies. To address these limitations, there is an urgent need for innovative delivery systems that enable precise targeting of immune cells while minimizing off‐target effects. In this study, we developed OncoLRC, a one‐component lipid‐RNA complex that enables efficient spleen‐targeted mRNA delivery and robust immune activation without the need for antibody modification. Our results demonstrate that OncoLRC robustly stimulates both innate and adaptive immune responses, marked by increased activation and expansion of splenic DCs, CD8^+^ T cells, and IFN‐γ^+^ T cells (Figure 4), underscoring its potential as a versatile cancer vaccine platform. Moreover, OncoLRC*^OVA^* elicited strong prophylactic and therapeutic antitumor responses in the B16F10‐OVA tumor model (Figures 5j and 6d).

“Cold tumors” characterized by poor infiltration of immune cells, particularly CD8^+^ T cells, often exhibit limited responsiveness to anti‐PD‐1/PD‐L1 monotherapy.^[^ ^62^, ^63^, ^64^ ^]^ To overcome this immunoresistance, various combination strategies are currently under investigation, including the addition of chemotherapy, anti‐angiogenic agents, DNA damage‐inducing agents (such as PARP inhibitors, cyclophosphamide, and/or radiotherapy), or other immune checkpoint inhibitors (e.g., CTLA‐4).^[^ ^65^ ^]^ Notably, integrating these therapies with immunotherapies like adoptive T cell therapy or cancer vaccines has emerged as a compelling strategy to sensitize cold tumors to immune attack.^[^ ^65^ ^]^ In this context, OncoLRC stands out due to its potent to robustly activate both innate and adaptive immunity, rendering it a strong candidate for combination therapies. While OncoLRC*^OVA^* monotherapy already conferred substantial tumor control, its combination with PD‐1 blockades further synergistically enhanced immune responses and prolonged survival in the B16F10‐OVA model (Figure 7b–d). This synergy is likely driven by alleviation of immunosuppression within the tumor, as evidenced by reduced proportions of PD‐1^+^ CD8^+^ T cells and Tregs in the TME (Figure 7n,o). These findings align with the growing body of evidence supporting rationally designed immunotherapy combinations,^[^ ^66^ ^]^ while offering a new insight into how a minimalist lipid‐mRNA complex can effectively complement immune checkpoint inhibitors.

Beyond efficacy, the favorable physicochemical and safety profiles of OncoLRC further support its translational promise. OncoLRC maintains colloidal stability and monodisperse structure at 4 °C (Figure S6a–c, Supporting Information), demonstrating its potential for addressing a key challenge in mRNA vaccine logistics. Unlike conventional LNPs that often incorporate PEG‐lipids, potentially eliciting anti‐PEG immune responses upon repeat dosing,^[^ ^49^, ^50^ ^]^ OncoLRC circumvents this issue through its PEG‐free, one‐component design. Additionally, the reduced lipid content in OncoLRC minimizes potential systemic toxicity risks, making it a promising candidate for clinical use.

## 4 Conclusion

In summary, our study introduces OncoLRC as a next‐generation mRNA vaccine delivery platform that addresses key limitations of *in‐situ* DC mRNA vaccine through spleen‐targeted delivery, robust immune activation, and simplified formulation. Its ability to reshape the tumor immune landscape and synergize with immune checkpoint blockade highlights its therapeutic versatility and potential to address difficult‐to‐treat “cold” tumors. Future studies should focus on elucidating the underlying mechanisms of its synergy with immunomodulatory agents. The OncoLRC technology holds great potential as a therapeutic platform for a wide array of disorders.

## 5 Experimental Section

### Cell Lines and Cell Culture

The B16‐OVA cell line is a murine B16F10 melanoma variant that expresses the chicken ovalbumin (OVA) gene, incorporating the H2‐Kb‐restricted OVA257‐264 epitope (SIINFEKL). This cell line was obtained from Prof. Jinjin Chen's lab at Sun Yat‐Sen University. The B16‐OVA cells were maintained in RPMI 1640 medium (HyClone), supplemented with 10% fetal bovine serum (FBS, Gibco) and 1% penicillin/streptomycin (Gibco). The DC2.4 cell line was obtained from RNACure Co., Ltd. (Shanghai, China), and cells were maintained in DMEM medium (Gibco), supplemented with 10% fetal bovine serum (FBS, Gibco) and 1% penicillin/streptomycin (Gibco).

### Animals

All animal experiments were conducted in accordance with protocols approved by the Fudan University Animal Care and Use Committee (approval number: 202207017S). Female C57BL/6 mice (6–8 weeks old) and female BALB/c mice (6–8 weeks old) were sourced from Lingchang Biotechnology Co., Ltd. (Shanghai). R26‐CAG‐LSL‐Luc‐tdTomato Cre‐reporter mice were acquired from Nanmo Organisms (Shanghai).

### Plasmid Construction for In Vitro Transcription

The OVA plasmid, which encodes the H‐2Kb‐restricted immunodominant epitope OVA257‐264, was obtained from Cynbio. The sequence of OVA257‐264 was amplified using the primers OVA_F (AGAGAACCCGCCACCATGGGCAGCATCGGCGC) and OVA_R (CGAGGCTCCAGCTCATCAGGGGCTCACGCACCG). The CAR‐hHER2‐luciferase plasmid was sourced from Aikangde Biotechnology Co., Ltd. (Suzhou). The CAR‐hHER2‐fluc sequence was amplified by PCR using primers CAR‐hHER2‐fluc_F(GAGAACCCGCCACCATGGCCCTCCCTGTCACCGC)and CAR‐hHER2‐fluc_R (CGAGGCTCCAGCTCATTACACGGCGATCTTTCCGCCCTTCTTGGCCTTTAT). The amplified DNA sequences were inserted into the IVT vector (Takara, 6143) with EcoRI/BamHI cutting sites using the Takara IVTpro mRNA synthesis system (Takara, 638 947). The recombinant plasmid was treated with Hind III restriction enzyme at 37 °C for 1 h, and the linearized template plasmid was purified using a PCR Purification Kit (Yeasen).

### In Vitro Transcription of mRNA

The fLuc mRNA was obtained from RNACure Co., Ltd. (Shanghai, China), and the circular RNA‐Luc was kindly provided by Prof. Liang Qu at Fudan University. Linearized template OVA plasmid was utilized for in vitro transcription (IVT) to synthesize mRNA. The mRNAs were generated via an IVT process employing T7 RNA Polymerase (Thermo Scientific), Pyrophosphatase (Thermo Scientific), RNase (Thermo Scientific), GTP (Synthgene), CTP (Synthgene), ATP (Synthgene), and N1‐Methylpseudouridine (N1‐Mep‐UTP, Synthgene). After transcription, the reaction mixture was treated with DNase I (Thermo Scientific), followed by precipitation and purification using lithium chloride (Thermo Scientific). To further isolate the mRNA, Oligo dT purification was performed, with Oligo dT sourced from Nanomicrotech Co., Ltd. (Suzhou). The quality of the synthesized mRNA was assessed using the Agilent 4200 Fragment Analyzer system.

### LNP Formulation and Characterization

For the initial LNP screening experiment, LNPs were prepared by adding the lipid mixture (the molar ratios of ionizable lipid: cholesterol: 1,2‐Distearoyl‐sn‐glycero‐3‐phosphorylcholine (DSPC): DMG‐PEG at 50: 38.5: 10: 1.5) in ethanol into the sodium acetate buffer (25 mM, pH 5.2) under vortex. Cholesterol, DSPC, and DMG‐PEG were purchased from AVT (Shanghai) Pharmaceutical Tech Co., Ltd. The obtained LNPs were then dialyzed using a Slide‐A‐Lyzer MINI Dialysis Device (3.5 K molecular weight cutoff, Thermo Scientific). We investigated the effect of different mass ratios of LNP to mRNA on spleen targeting. LNP/mRNA formulations were prepared by simply mixing blank LNPs and mRNA at mass ratios ranging from 10:1 to 3:1 (ionizable lipid/mRNA). For LNP formulation optimization, a systematic Design of Experiments (DOE) approach was applied. A two‐factor, three‐level fractional factorial design was constructed using Minitab software to efficiently screen formulation variables while minimizing experimental runs. To dissect the role of each structural component, it was first evaluated three‐component LNP formulations by selectively excluding cholesterol or DSPC, or DMG‐PEG2000 from the canonical four‐component formulation. The physicochemical properties of these formulation matrices are presented in Figure S3a,c,e (Supporting Information), corresponding to the exclusion of cholesterol, DMG‐PEG2000, and DSPC, respectively. For the two‐component formulations, the ionizable lipid: cholesterol or ionizable lipid: DSPC (mass ratio), ranging from 5:1 to 1:5 was investigated. Under the single‐component formulation condition, the lipid/mRNA mass ratios ranging from 15:1 to 1.5:2 was investigated. The size and polydispersity index (PDI) of LNPs were characterized using a Zetasizer Nano (Malvern Instruments, Malvern). For in vivo screening, 5 µg of LNP‐RNA per mouse was administered by intravenous injection. For the stability studies, freshly prepared OncoLRC*^Luc^* was stored at 4 °C for 1–10 days before injection. For immunological and tumor experiments, mice were immunized three times with 20 µg OncoLRC*^OVA^* unless stated otherwise. The generation of memory T cells was verified by the recall response one week after the final immunization. Control groups received either PBS or OncoLRC*^irrelevant mRNA^*.

LPX was prepared using the thin‐film hydration method as previously described.^[^ ^41^ ^]^ Briefly, stock solutions of the cationic lipid N‐ [1‐(2, 3‐dioleyloxy) propyl]‐ N, N, *N*‐trimethylammonium chloride (DOTMA) and dioleoylphosphatidylethanolamine (DOPE) were dissolved in dichloromethane at 10 mg mL^−1^. The two lipids were mixed at a DOTMA: DOPE molar ratio of 1:1 and dried using a rotary evaporator at 40 °C and 10 rpm for 2 h to form a thin lipid film. The dry film was subsequently hydrated with RNase‐free water under gentle agitation. The resulting crude liposome suspension was sonicated for 15 min (30 s on/ 30 s off cycles), followed by incubation at 4 °C overnight to allow equilibration.

### Apparent pKa Measurement

Using a fluorescence‐based method with 2‐(p‐toluidinyl)‐6‐naphthalenesulfonic acid (TNS), the *p*Ka values of various active lipids in lipid nanoparticles were determined. TNS was prepared as a 300 µM stock solution in dimethyl sulfoxide (DMSO). The pH buffer solutions contained 10 mM sodium phosphate, 10 mM sodium borate, 10 mM sodium citrate, and 150 mM sodium chloride, with the pH adjusted to 2, 3, 4, 5, 5.5, 6, 6.4, 6.8, 7, 7.5, 8, 8.5, 9, 10, 11, and 12 using 2 M sodium hydroxide and 2 M hydrochloric acid. In a black, clear‐bottomed 96‐well plate, 94 µL of each pH buffer solution was added, followed by 2 µL of the 300 µM TNS solution, and then 4 µL of the lipid nanoparticle composition. After standing at room temperature for 5 min, the fluorescence intensity was measured using an enzyme‐linked immunosorbent assay (ELISA) reader (Thermo Fisher) with excitation wavelengths of 325 nm and emission wavelengths of 435 nm. The fluorescence data were analyzed using sigmoid curve fitting, and the *p*Ka was defined as the pH value at which half‐maximal fluorescence intensity was achieved.

### In Vivo mRNA Delivery

For in vivo imaging studies, Balb/c mice (6‐8 weeks old) were intravenously injected with LNPs containing either 5 µg of fLuc mRNA, or 100 µg of CAR‐hHER2‐Luc mRNA, or 100 µg of circular RNA‐Luc per mouse. Six hours post‐injection, 100 µL of D‐luciferin at a concentration of 30 mg mL^−1^ in PBS was intraperitoneally injected into the mice. Ten minutes later, the mice were imaged using an in vivo imaging system (IVIS). For the repeated dosing study, mice were intravenously administered fLuc mRNA (0.25 mg kg^−1^, *n* = 3) on days 0, 3, 8, 13, and 18, and imaged at 6 h after each injection. To further identify the cell types transfected in vivo, we injected OncoLRC*^Cre^* (3 mg/kg) into the R26‐CAG‐LSL‐Luc‐tdTomato Cre‐reporter mice. The mice were euthanized 5 days after injection, and spleen and bone marrow cells were collected. The cells were stained with anti‐CD45‐APC‐Cy7, anti‐CD3‐PerCP‐Cy5.5, anti‐CD4‐ Alexa Fluor 700, anti‐CD8‐BV605, anti‐NK1.1‐APC, anti‐CD11c‐BV605, anti‐CD11b‐BV650, anti‐F4/80‐FITC, anti‐CD317‐APC, and anti‐CD19‐APC/Fire 810. See the FACS section for specific sample processing and staining methods. Gating information shown in Figures S8 and S9 (Supporting Information). Representative diagrams of flow cytometry analysis were shown in Figures S8 and S9 (Supporting Information).

### Flow Cytometry

Antibodies used in this study are listed in Table S3 (Supporting Information).

### Spleen Cells

To isolate and stain splenic cells, the spleen was first placed into a 70 µm cell strainer (Biologix) and positioned in one well of a six‐well plate. Two milliliters of DMEM culture medium were then added to the well, and the spleen was mechanically ground using the plunger of a 1 mL syringe on ice. The resulting cell suspension was transferred to a 2 ml EP tube and centrifuged at 400 × g for 5 min at 4 °C. The pellet was resuspended in 2 mL of ice‐cold RBC lysis buffer (Sangon Biotech, Shanghai) and incubated on ice for 5 min to lyse red blood cells. To terminate the lysis, 2 ml of DMEM was added, followed by another round of centrifugation at 400 × g for 5 min. The cell pellet was then washed three times with staining buffer (PBS + 2% FBS), followed by blocking with anti‐CD16/32 (Biolegend) for 15 min to prevent non‐specific binding. Afterward, cells were stained with surface antibodies (final volume: 100 µL) for 30 min in the dark at 4 °C. The stained cells were washed twice with 1 ml of staining buffer, and FACS data were acquired using the Cytek Aurora cytometer (5‐laser configuration). Finally, the data analysis was conducted using FlowJo software.

### Bone Marrow Cells

Following euthanasia, femurs and tibias were aseptically harvested from C57BL/6 mice and immediately immersed in 70% ethanol for 1 min for surface sterilization. The epiphyseal ends were surgically excised at the ankle joint level using sterile instruments. Marrow cavities were flushed with chilled DMEM medium (Gibco) using a 25‐gauge needle until complete bone blanching was achieved. The cell suspension was sequentially filtered through a 70‐µm nylon mesh (Corning) to eliminate bone fragments and tissue debris, followed by centrifugation at 250 × g for 5 min at 4 °C. Erythrocyte depletion was performed by resuspending the pellet in 2 mL ice‐cold RBC lysis buffer (Sangon Biotech, Shanghai) for precisely 5 min on ice, with subsequent neutralization using 5 mL complete DMEM medium. The bone marrow cells were collected by centrifugation (250 × g, 5 min) and resuspended in staining buffer for downstream applications. Surface antigen labeling was performed following the same methodology applied to splenocytes. FACS data were acquired on the Cytek Aurora cytometer (5‐laser configuration), and data analysis was conducted in FlowJo software.

### Peripheral Blood Cells

Peripheral blood (100 µL) was collected from the murine retro‐orbital venous plexus using glass capillaries (Kimble Chase) and immediately transferred to EDTA‐coated tubes to prevent coagulation. For erythrocyte depletion, blood samples were treated with 2 mL ice‐cold RBC lysis buffer (Sangon Biotech, Shanghai) with gentle vortexing, followed by 20 min incubation on ice with periodic mixing. The lysate was centrifuged at 400 × g for 5 min at 4 °C, and the leukocyte‐rich pellet was washed twice with 2 mL cold DMEM supplemented with 10% FBS (Gibco). When residual erythrocytes were observed, the lysis procedure was repeated for 5 min. The isolated cells were processed through a standardized immunostaining protocol beginning with a 15 min blocking step using anti‐CD16/32 (clone 2.4G2) at 4 °C to minimize nonspecific binding, followed by surface antigen labeling with titrated fluorochrome‐conjugated antibodies in 100 µL of staining buffer (PBS containing 2% FBS) for 30 min at 4 °C. For intracellular marker detection, cells were washed twice with BD Perm/Wash Buffer (Cat# 554 723) before fixation and permeabilization with BD Cytofix/Cytoperm solution (20 min, 4 °C), then incubated with pre‐optimized intracellular antibodies for 30 min at room temperature under light‐protected conditions. Finally, cells were resuspended in 250 µL of FACS buffer and samples were acquired on the Cytek Aurora cytometer (5‐laser configuration). Then the data analysis was conducted using FlowJo software.

### Tumor Cells

To generate single‐cell suspensions from solid tumors, tissue samples were mechanically dissociated using the DSC‐400 Single Cell Suspension Dissociator (RWD Life Science, Shenzhen, China). The resulting cell suspension was sequentially filtered through a 70‐µm cell strainer (Corning) to remove undigested tissue fragments and centrifuged at 400 × g for 5 min at 4 °C. Erythrocyte contamination was eliminated by incubating the pellet with 2 mL of pre‐chilled RBC lysis buffer (Sangon Biotech, Shanghai) for precisely 5 min on ice, followed by quenching with an equal volume of complete DMEM medium (Gibco) supplemented with 10% FBS. After centrifugation (400 × g, 5 min), the leukocyte‐enriched fraction was isolated and subsequently washed three times with ice‐cold staining buffer. For immunophenotyping, both surface and intracellular staining procedures were performed following the standardized protocol used for peripheral blood cells. Specifically, nuclear permeabilization was conducted using the eBioscience Foxp3/Transcription Factor Staining Buffer Set (Invitrogen, 00‐5523‐00), according to the manufacturer's instructions. Following permeabilization, cells were incubated with the target antibodies in 100 µL of staining buffer for 30 min at room temperature, protected from light. After staining, cells were washed twice with 1 mL of staining buffer to remove unbound antibodies. Finally, the labeled cells were resuspended in 250 µL of staining buffer and analyzed immediately on the Cytek Aurora cytometer (5‐laser configuration). Then the data analysis was conducted using FlowJo software.

### ELISA for IFN‐α, IL‐12p40, and IFN‐γ

Serum samples were collected at 6 and 24 h post‐intravenous administration of 20 µg OncoLRC*^OVA^* to 6‐week‐old C57/BL6 mice (*n* = 4). Cytokine levels in serum were quantified using commercial ELISA kits according to manufacturers' protocols: IFN‐α was measured using the ELISA MAX Deluxe Set Mouse IFN‐α kit (BioLegend, 447 904); IL‐12p40 was analyzed with the Mouse IL‐12/IL‐23p40 ELISA Kit (Lianke Bio, Hangzhou, EK2183); and IFN‐γ was assessed using the Mouse IFN‐γ ELISA Kit (Lianke Bio, Hangzhou, EK280HS).

### ELISA for Antibody Titer

The antibody titer was measured by indirect ELISA. The high‐binding ELISA plates (Thermo Scientific) were covered with 100 µL of OVA at 10 ug mL^−1^ in PBS at 4 °C for 20 h. The plates were then washed with PBS containing 0.5% Tween‐20 and blocked with 2% bovine serum albumin solution (Sigma–Aldrich). The serum collected from immunized mice was initially diluted 1:100. After performing a serial dilution in quadruplicate, the diluted serum was added to the plates for 2 h at 37 °C. Then the plates were washed and incubated with peroxidase‐conjugated anti‐IgG, IgG1, and IgG2c antibodies for 1 h. The plates were washed and incubated with 100 µL of 3,30,5,50‐tetramethylbenzidine substrate (Sigma–Aldrich, T8665‐100ML). The reaction was stopped by the stop solution (Adamas, C8304‐100 mL). The optical density at 450 nm was measured by a Multiskan FC microplate reader (Thermo Scientific). The endpoint titer was defined as the reciprocal of the highest dilution of a serum that gives a reading above the cutoff (two times the PBS group).

### Enzyme‐Linked immunospot (ELISpot) Assay

Five days after the third vaccination, spleen cells were isolated and suspended in 200 µL of RPMI‐1640 medium containing 10% fetal bovine serum. The ELISpot assay was performed using the Mouse Interferon Gamma ELISPOT Kit (ab64029, Abcam, USA). A total of 1 × 10⁶ spleen cells were incubated in complete RPMI‐1640 medium, with or without 2 µg mL^−1^ of SIINFEKL peptide (GenScript, RP1066‐CN), at 37 °C for 18 h. After incubation, the plates were washed and incubated with biotinylated anti‐IFN‐γ antibody, followed by streptavidin‐alkaline phosphatase conjugate, according to the manufacturer's protocol. Images were captured, and the number of spots for each mouse was automatically calculated.

### Prophylactic Efficacy in OncoLRC*^OVA^* Immunized C57BL/6

C57BL/6 mice (6‐8 weeks old) were immunized repetitively with 20 µg OncoLRC*^OVA^* on day 0, day 3, and day 8. Seven days after the last immunization, 5 × 10^5^ B16F10‐OVA tumor cells were inoculated subcutaneously into the flanks of mice. The length (L) and width (W) of the mice's tumors were measured every other day. The tumor volume (V) was calculated using the formula: V = (L × W^2)/2.

Animals were euthanized when exhibiting signs of impaired health or when the volume of the tumor exceeded 1500 mm^3^.

### OncoLRC*^OVA^* for Subcutaneous B16F10‐OVA Solid Tumor Therapy

To establish the B16F10‐OVA tumor model, 5 × 10^5^ of B16‐OVA cells were injected subcutaneously into the right flank of C57BL/6 (6‐8 weeks old) on day 0. On day 9, mice were divided into three groups: OncoLRC*^OVA^* treatment group, OncoLRC*^Luc^* control group, and untreated control group. Mice in the OncoLRC*^OVA^* and OncoLRC*^Luc^* groups received nanomedicine on days 9, 12, and 17, with a dosage of 20 µg of OVA mRNA or fluc mRNA per injection, respectively. Tumor sizes were measured unblinded with a caliper every other day for calculating tumor volumes using the equation (L × W^2)/2 (L, length; W, width). Animals were euthanized when exhibiting signs of impaired health or when the volume of the tumor exceeded 1500 mm^3^. On day 24, the peripheral blood of the mice was analyzed by flow cytometry. On day 25, the mice were euthanized, and the tumors were collected and processed into single‐cell suspensions for further flow cytometry analysis. Representative flow cytometry diagrams are shown in Figures S13 and S14 (Supporting Information).

### OncoLRC*^OVA^* Combined with aPD‐1 Treatment

The establishment of the B16F10‐OVA tumor model and the treatment schedule for OncoLRC*^OVA^* are the same as those described in the OncoLRC*^OVA^* for subcutaneous B16F10‐hHER2 solid tumor therapy' section. On days 11, 15, and 19, aPD‐1 (100 µg mouse^−1^) was administered intraperitoneally as a combination treatment group, while the group receiving only OncoLRC*^OVA^* did not receive the injection. On day 24, the mice were euthanized, and the spleens and tumors were collected and processed into single‐cell suspensions for further flow cytometry analysis. Representative flow cytometry diagrams are shown in Figure S16 (Supporting Information).

### Statistical Analysis

All experiments were conducted in three independent replicates. Statistical analysis was performed using GraphPad Prism (version 9.5) software. Quantitative results were expressed as mean ± SD. A two‐group comparison was conducted using the unpaired two‐tailed Student's t test. Multiple groups and/or multiple condition comparisons were conducted using one‐way or two‐way analysis of variance (ANOVA). Overall survival was analyzed with Kaplan‐Meier curves, and differences in survival among subgroups were analyzed using the log‐rank test. Statistical significance was defined as *p*<0.05, *p<0.05, **p<0.01, ***p<0.001, and ****p<0.0001 in figures and figure legends.

## Conflict of Interest

The authors declare no conflict of interest.

## Supporting information



Supporting Information

## Data Availability

Research data are not shared.
