# Supplementary material for: A Rationally Engineered Spleen‐Tropic One‐Component Lipid‐mRNA Complex (OncoLRC) for Cancer Vaccines
Source: Adv Sci (Weinh). 2025 Nov 21;13(8):e12535. doi: 10.1002/advs.202512535 (PMC12884810; doi:10.1002/advs.202512535)
Supplement: Supplementary file 1 — Supporting Information [file ADVS-13-e12535-s001.doc]

**Supplementary information**

**A rationally engineered spleen-tropic one-component lipid-mRNA complex (OncoLRC) for cancer vaccines**

*Qimeng Yin, Chenchen Zhang, Jiahao Li, Kun Huang, and Min Qiu**

1. Yin, C. Zhang, J. Li, K. Huang, M. Qiu

Human Phenome Institute, Fudan University, Shanghai 201203, China

M. Qiu

Center for mRNA Translational Research, Fudan University, Shanghai 200438, China

Zhangjiang mRNA Innovation and Translation Center, Shanghai 201203, China
E-mail: mqiu@fudan.edu.cn (M. Q.).

**Table of content**

**Supplementary Figures ................................................................................2**

**Supplementary Tables ..................................................................................15**


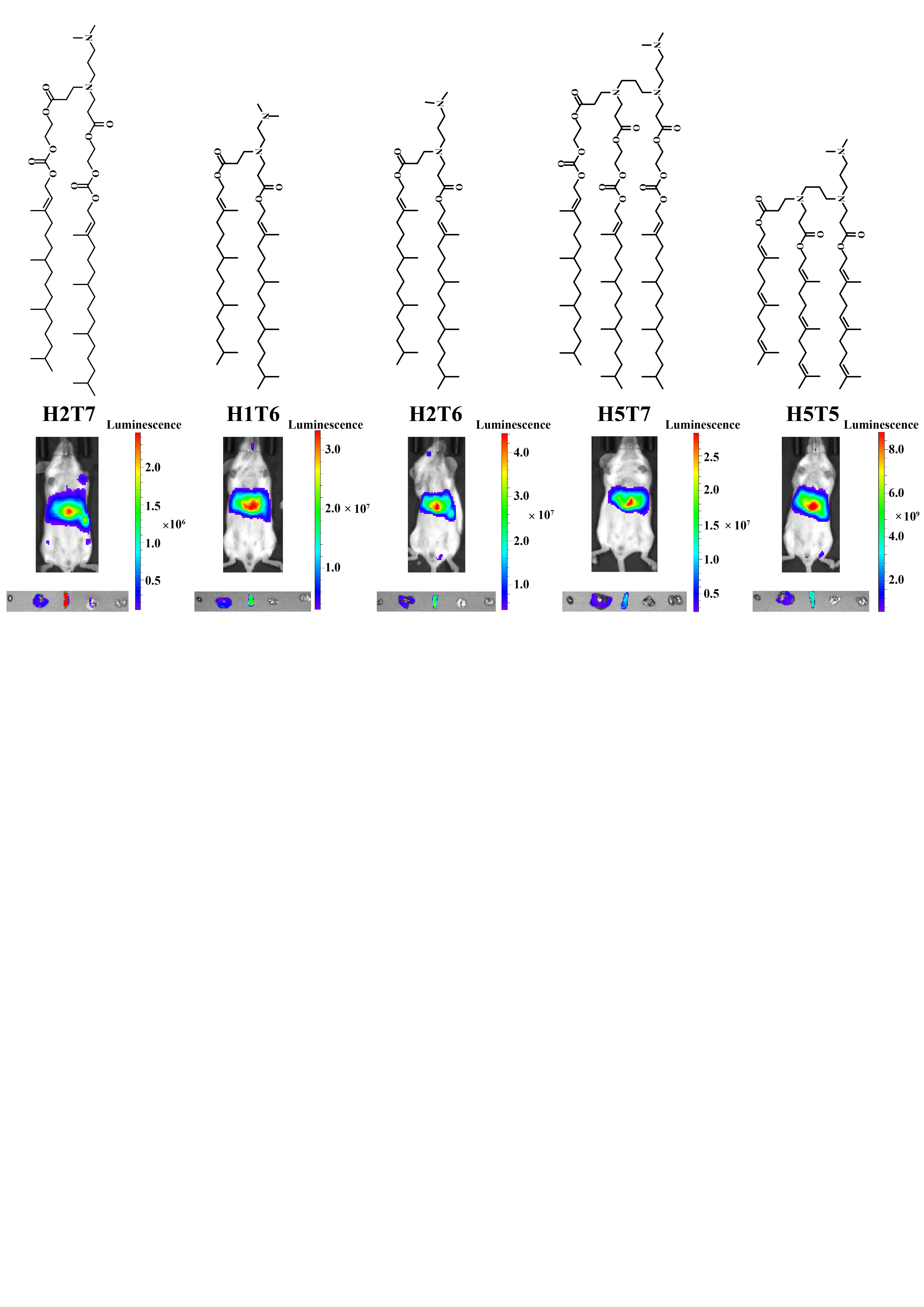


**Supplementary Figure 1.** Representative in vivo bioluminescence images of the DMA-lipidoids.


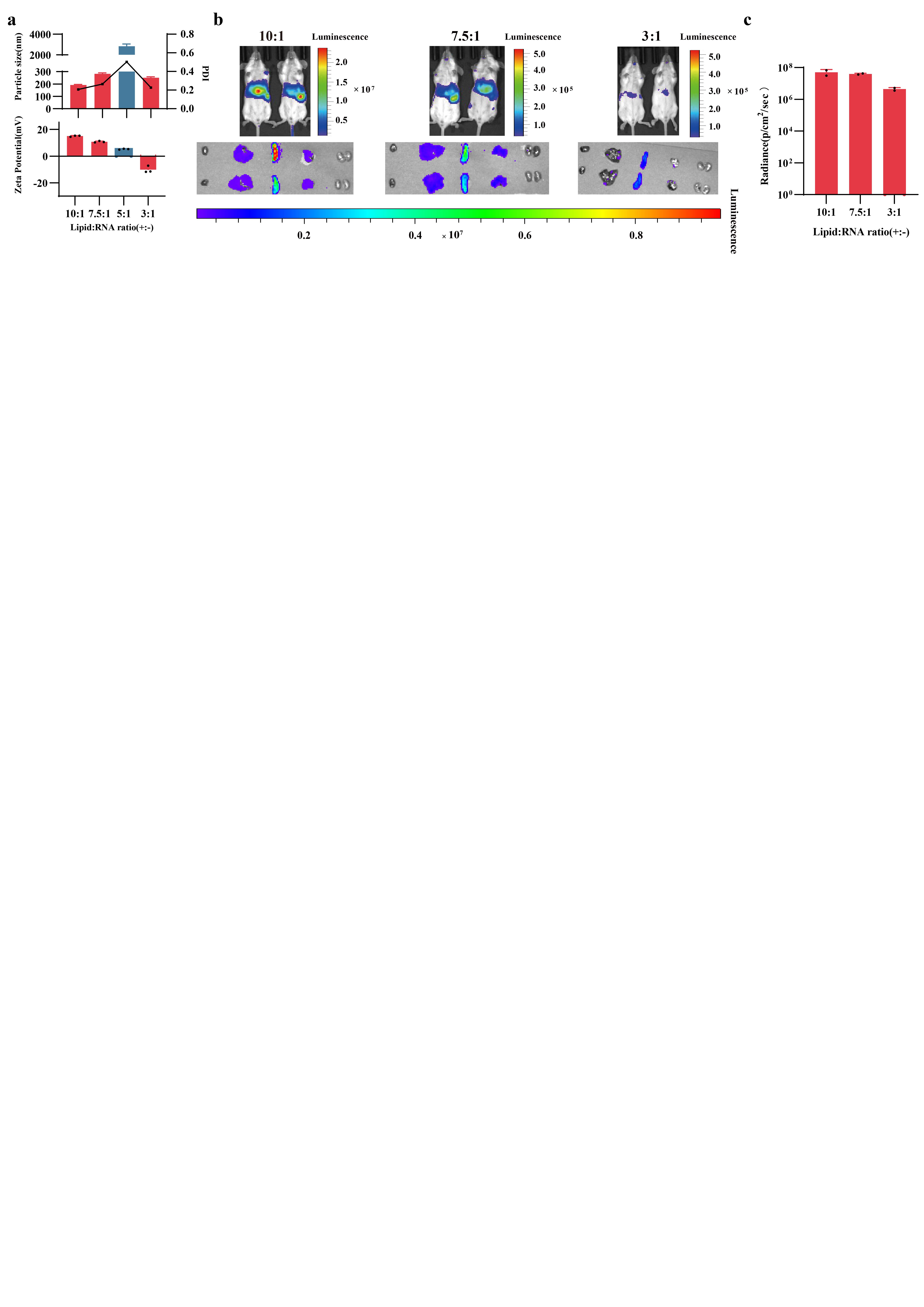


**Supplementary Figure 2. Investigation of the relationship between the mass ratio of four-component (standard formulation) H2T7 LNP to mRNA and spleen targeting.** (a) Particle size, polydispersity index (top), and zeta potential (bottom) of the standard formulation complexed with Luc mRNA at various mass ratios (n = 3). (b) Bioluminescence imaging of BALB/c mice (n = 2) 6 hours after intravenous (*i.v.*) injection of LNP-Luc at various mass ratios. (c) Quantification of whole-body bioluminescence intensity 6 hours post-treatment with LNP-Luc (n = 2). Data are presented as mean ± SD.


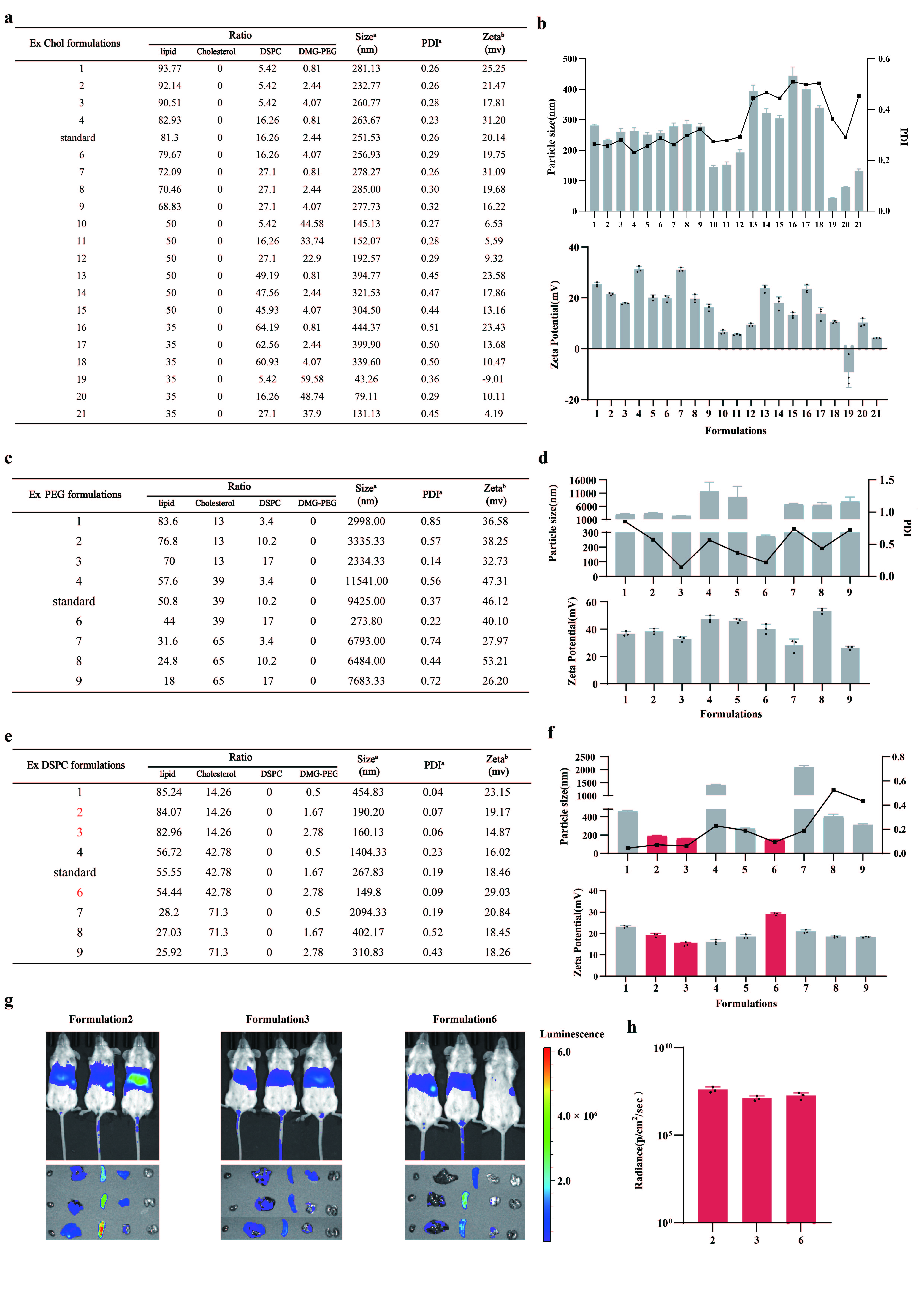


**Supplementary Figure 3. Optimization of three-component formulations.** (a) DOE-designed 21 formulations (cholesterol removed) and their corresponding particle size, polydispersity index, and zeta potential. (b) Bar plots of particle size, polydispersity index, and zeta potential for the 21 DOE-designed formulations (cholesterol removed). (c) DOE-designed 9 formulations (PEG removed) and their corresponding particle size, polydispersity index, and zeta potential. (d) Bar plots of particle size, polydispersity index, and zeta potential for the 9 DOE-designed formulations (PEG removed). (e) DOE-designed 9 formulations (DSPC removed) and their corresponding particle size, polydispersity index, and zeta potential. (f) Bar plots of particle size, polydispersity index, and zeta potential for the 9 DOE-designed formulations (DSPC removed). (g) Bioluminescence imaging of BALB/c mice (n = 3.) 6 hours after intravenous (*i.v.*) injection of three stable formulations encapsulating Luc mRNA. (h) Quantification of whole-body bioluminescence intensity 6 hours post-treatment with three stable formulations encapsulating Luc mRNA (n = 3). Data are presented as mean ± SD.


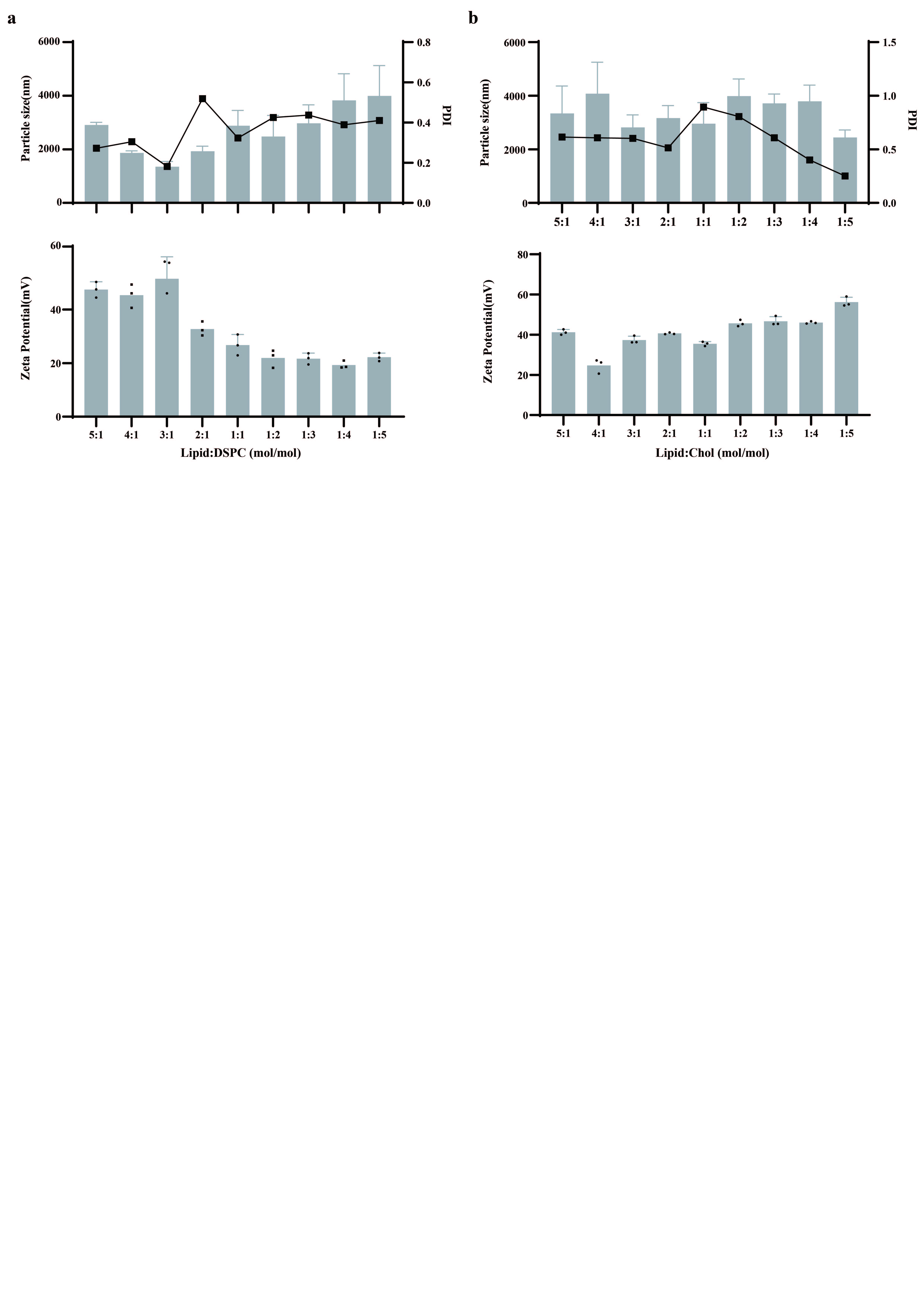


**Supplementary Figure 4. Optimization of two-component formulations.** (a) Particle size, polydispersity index (top), and zeta potential (bottom) of the two-component formulations complexed with Luc mRNA at various molar ratios of H2T7 to DSPC (n = 3). (b) Particle size, polydispersity index (top), and zeta potential (bottom) of the two-component formulations complexed with Luc mRNA at various molar ratios of H2T7 to cholesterol (n = 3). Data are presented as mean ± SD.


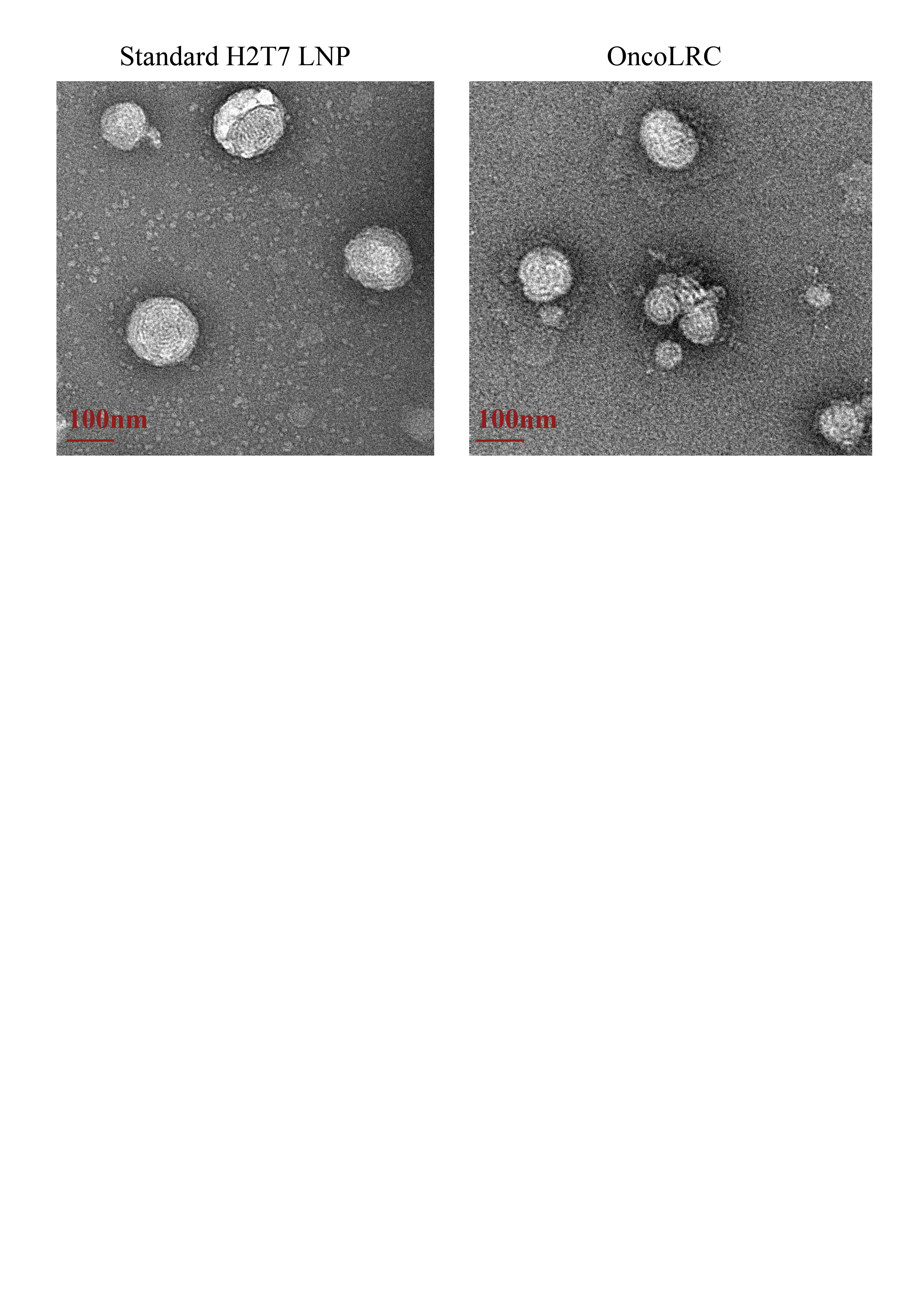


**Supplementary Figure 5.** Negative stain electron micrographs of (left) standard H2T7 LNP-Luc formulated at an active lipid/mRNA mass ratio of 10:1 and (right) OncoLRC*^Luc^* formulated at a lipid/mRNA mass ratio of 1.5:1. Scale bar: 100 nm.


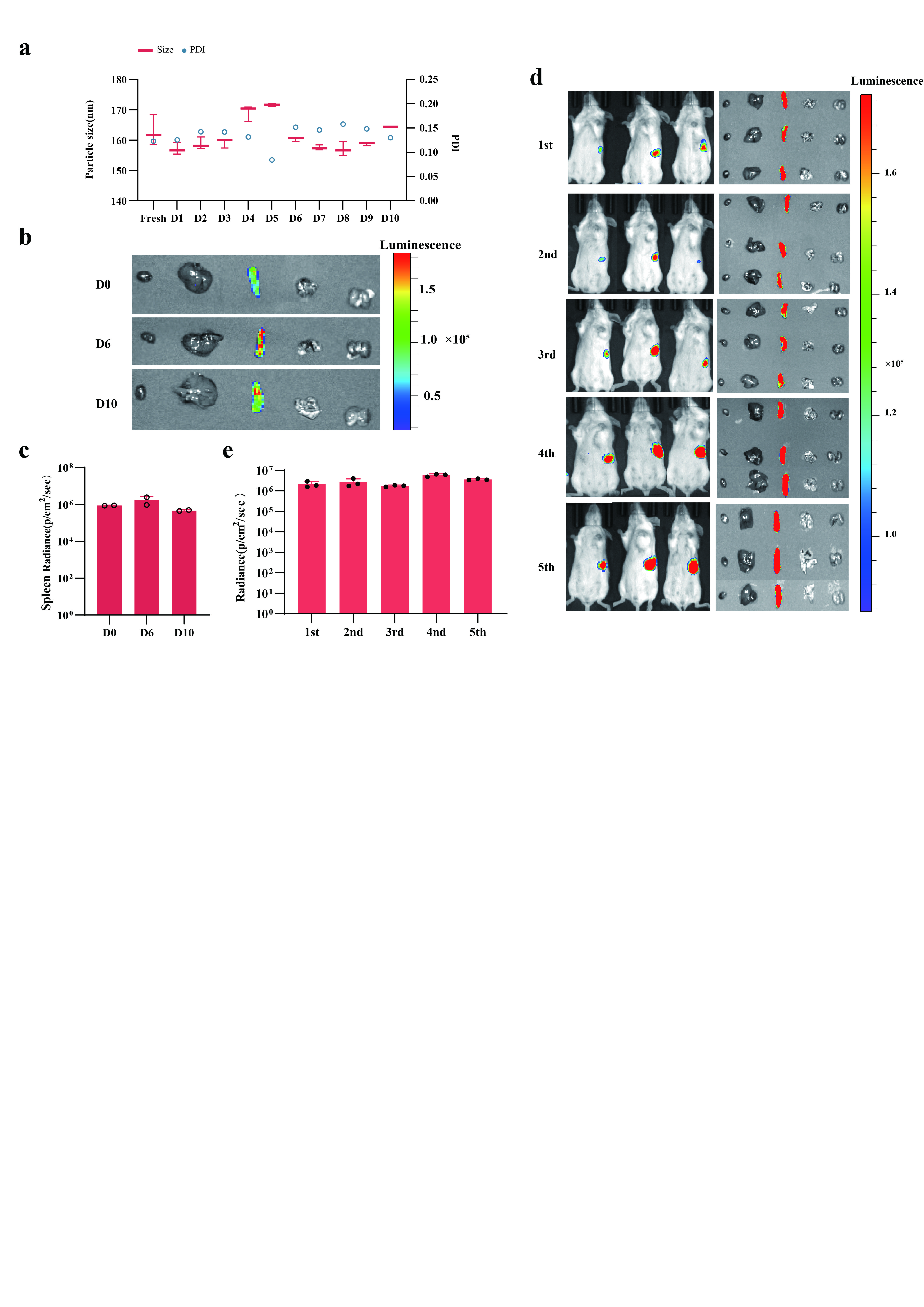


Supplementary Figure 6. (a) Changes in size and polydispersity index of OncoLRC*^Luc^* during the storage at 4 °C. Prior to measurement, samples were removed from storage and equilibrated at room temperature for 24 h (n = 3). Representative bioluminescence images (b) and corresponding quantification of splenic signal intensities (c) demonstrating the spleen-selective distribution of OncoLRC*^Luc^* following intravenous (*i.v.*) injection, measured at different storage time points (n = 2). Representative *in vivo* and *ex vivo* IVIS bioluminescence images (d) and the corresponding quantification of splenic signal intensities (e) following five repeated administrations, captured 6 h after each *i.v.* injection of fLuc mRNA (0.25 mg/kg, n = 3). Data are presented as mean ± SD.


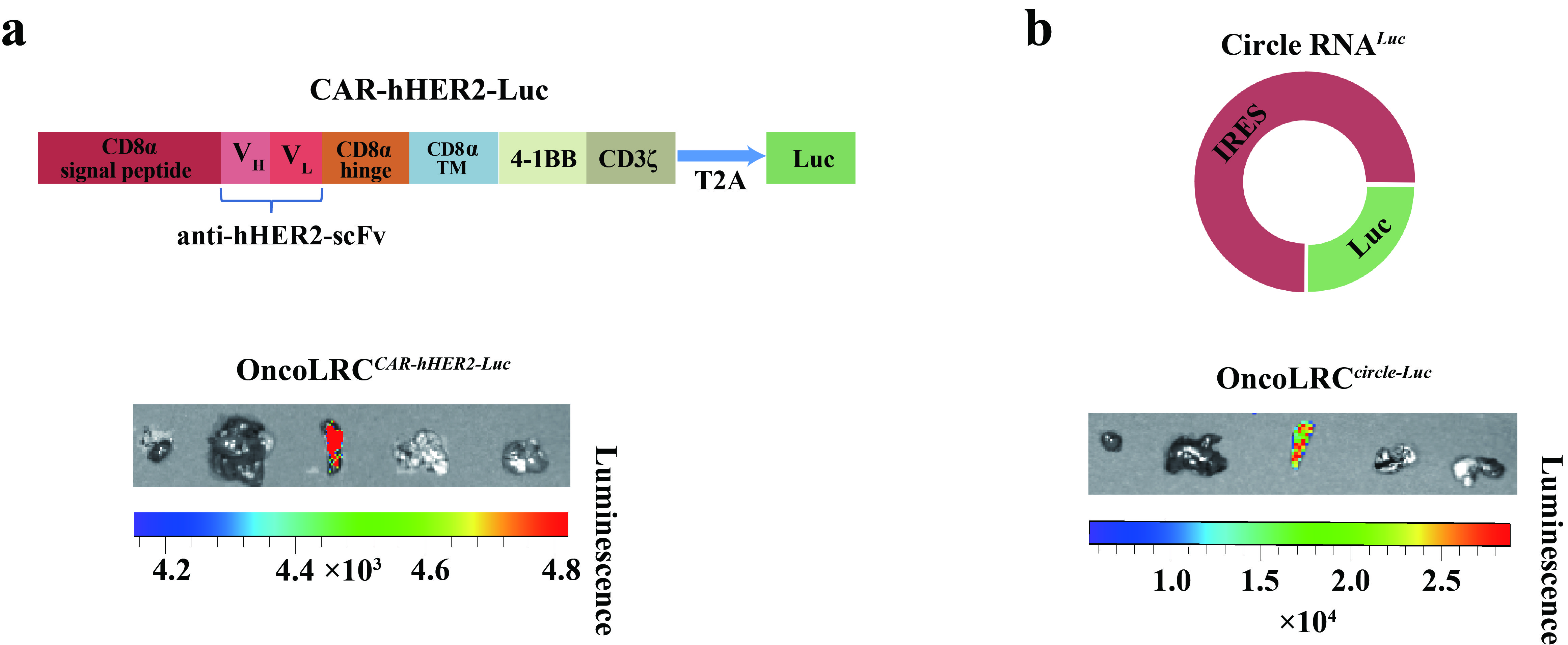


Supplementary Figure 7. Bioluminescence images showing spleen-selective delivery of CAR-hHER2-Luc mRNA (a) and circular RNA-Luc (b) by OncoLRC following *i.v.* injection. Images were taken 6 h post-administration (5mg/kg, n = 2).


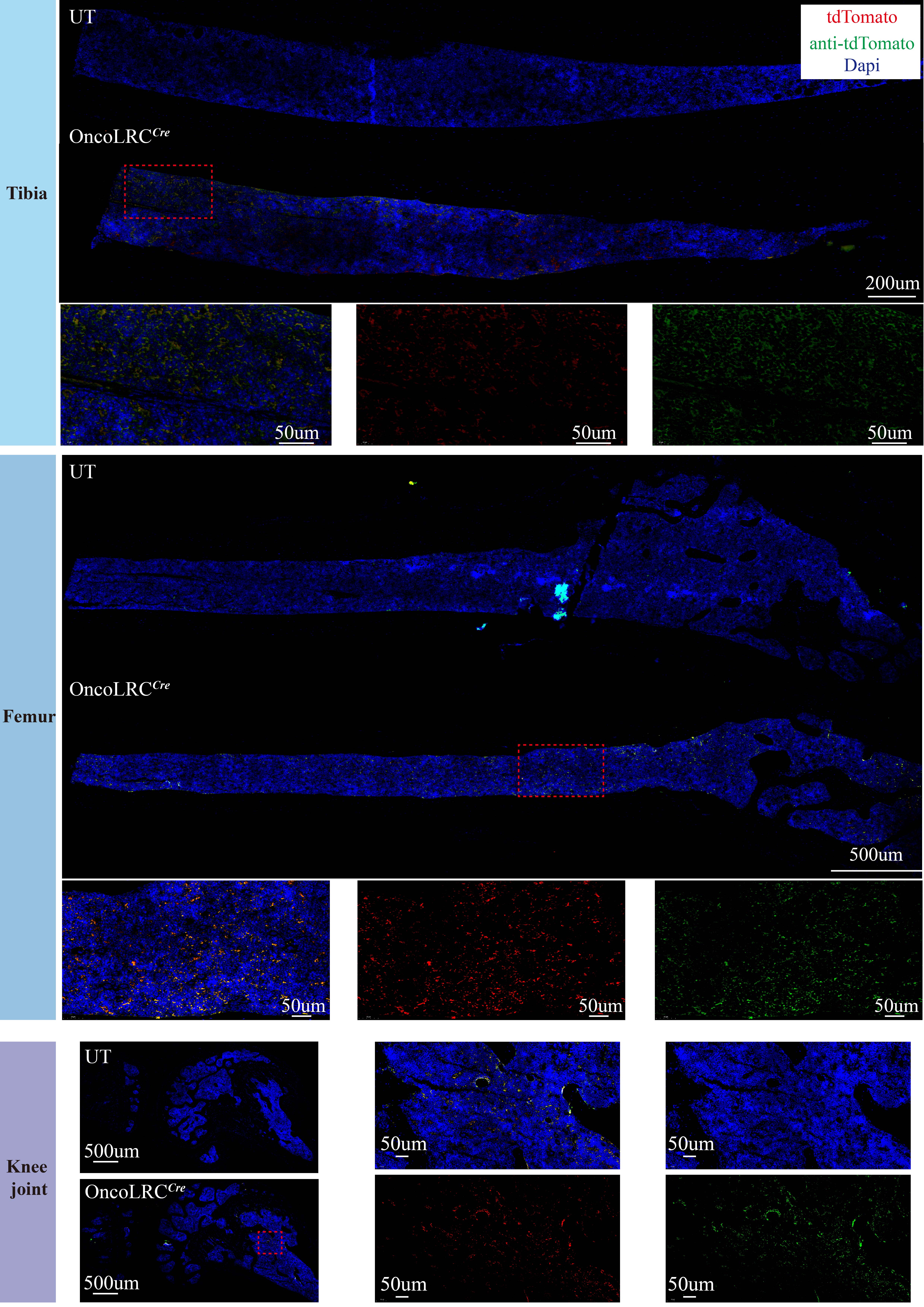


**Supplementary Figure 8.** Immunofluorescence staining of the tibia, femur, and knee joint sections from mice treated with OncoLRC*^Cre^*. Insets show magnified views of the regions outlined by red dashed boxes.


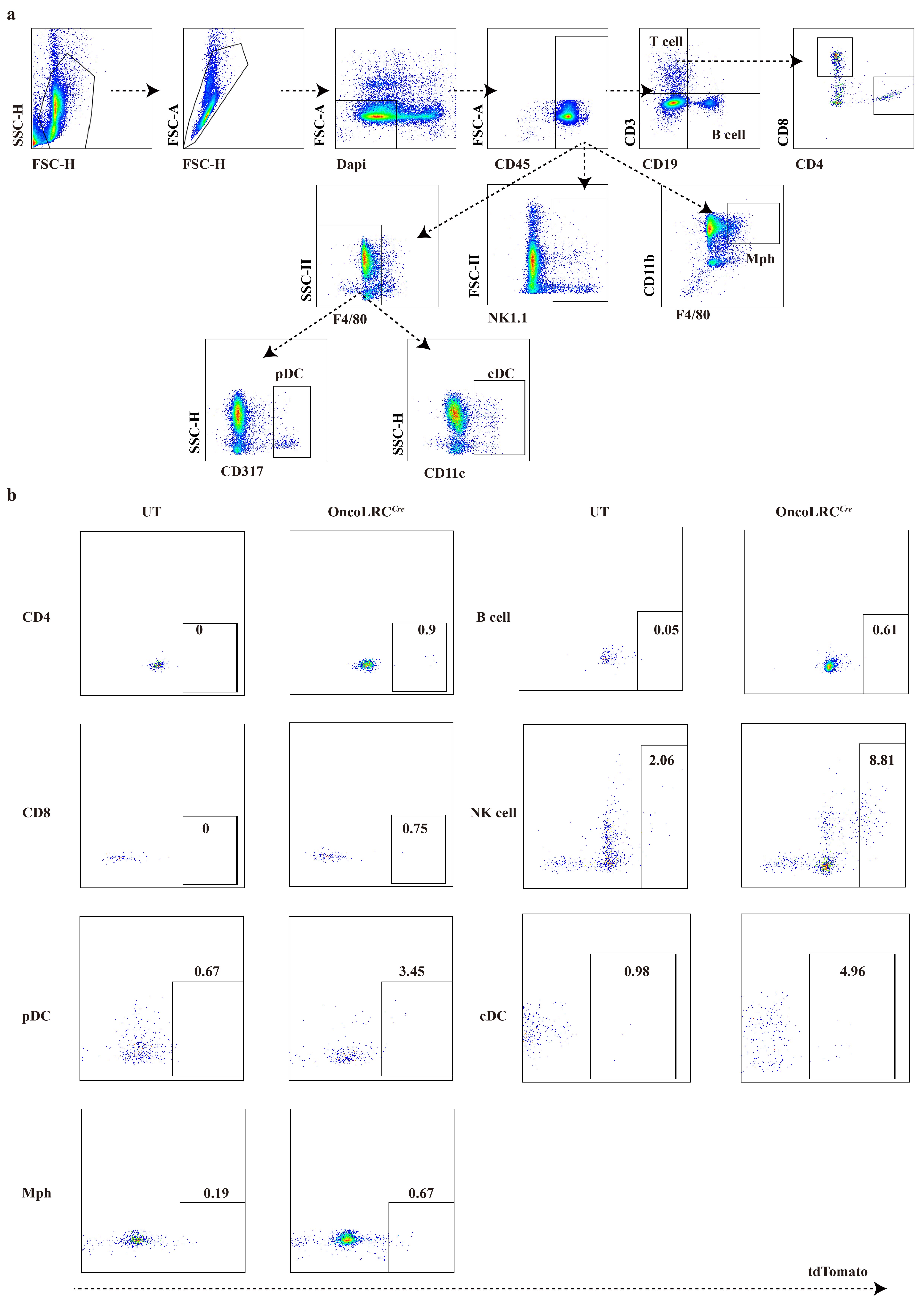


**Supplementary Figure 9.** (a) Gating strategy used for flow cytometry of immune cells in bone marrow of mice treated with OncoLRC*^Cre^*. (b) Representative FACS plots showing tdTomato-positive cells in the bone marrow.


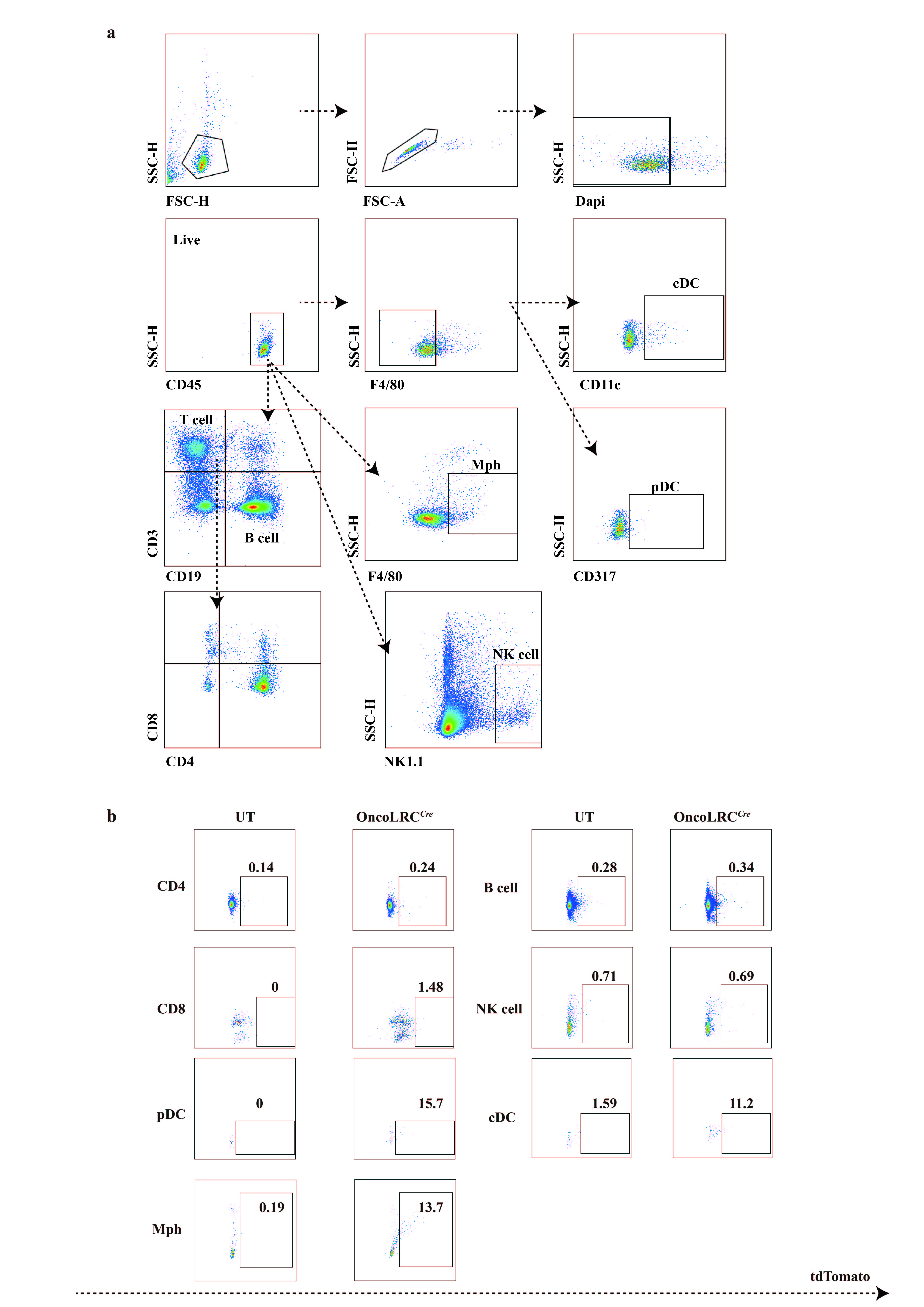


**Supplementary Figure 10.** (a) Gating strategy used for flow cytometry of immune cells in spleen of mice treated with OncoLRC*^Cre^*. (b) Representative FACS plots showing tdTomato-positive cells in the spleen.


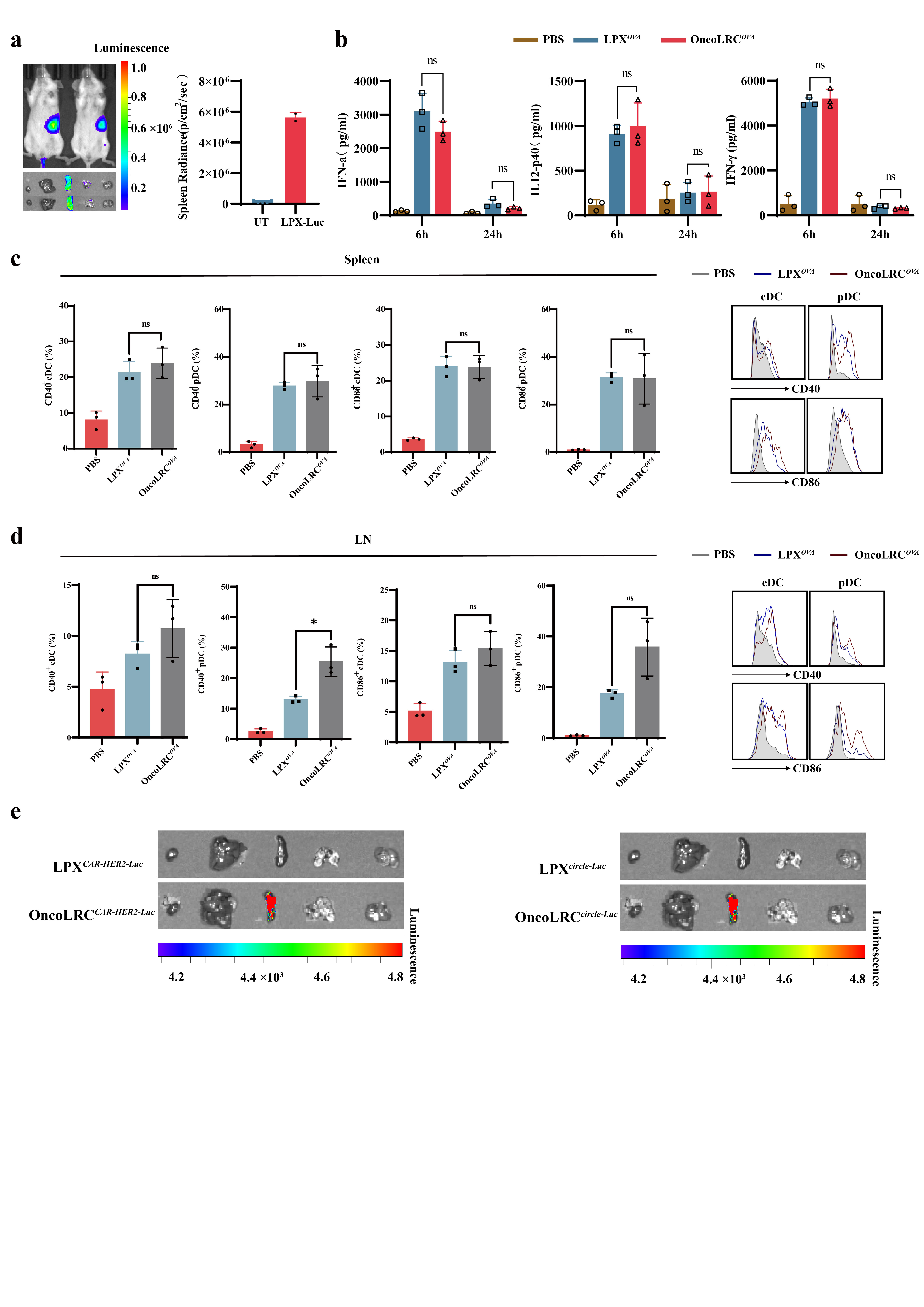


**Supplementary Figure 11.** (a) Bioluminescence images and corresponding quantification of splenic signal intensities 6 h after *i.v.* injection of LPX-Luc (1mg/kg, n = 2). (b) Serum levels of IFN-α, IL-12p40, and IFN-γ in C57BL/6 mice at 6 and 24 h after *i.v.* injection of OncoLRC*^OVA^* or LPX*^OVA^*. Percentage of CD40^+^ and CD86^+^ pDCs and cDCs in spleen (c) and lymph node (d) 24 h after *i.v.* injection of OncoLRC*^OVA^* or LPX*^OVA^* (n = 3). Significance was determined using one-way ANOVA and Tukey’s multiple comparisons test (b, c, d). Data are presented as mean ± SD. **P* < 0.05, ***P* < 0.01, ****P* < 0.001, *****P* < 0.0001.


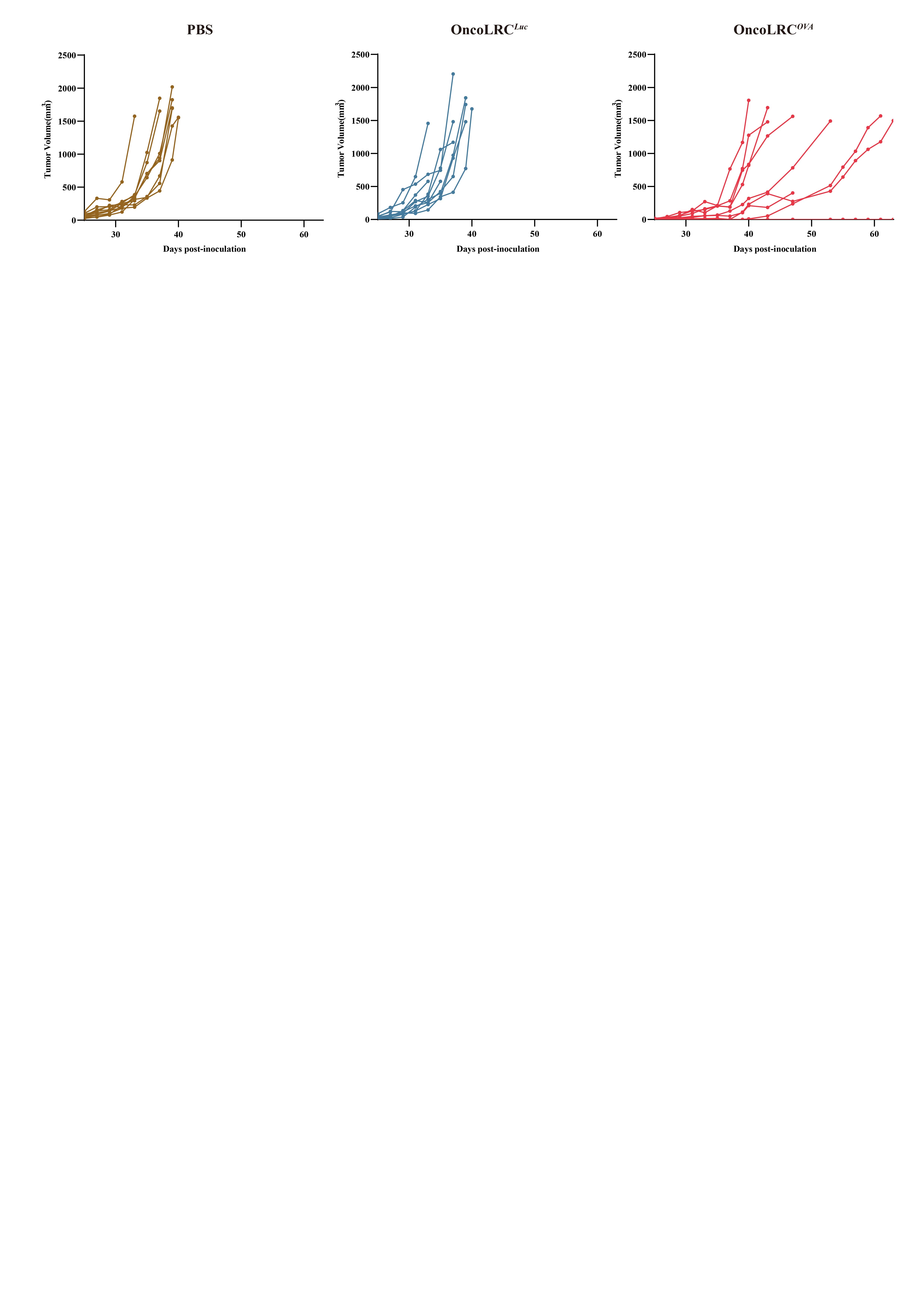


**Supplementary Figure 12.** Tumor growth profiles of mice in different treatment groups in the protective immunity experiment (n = 10).


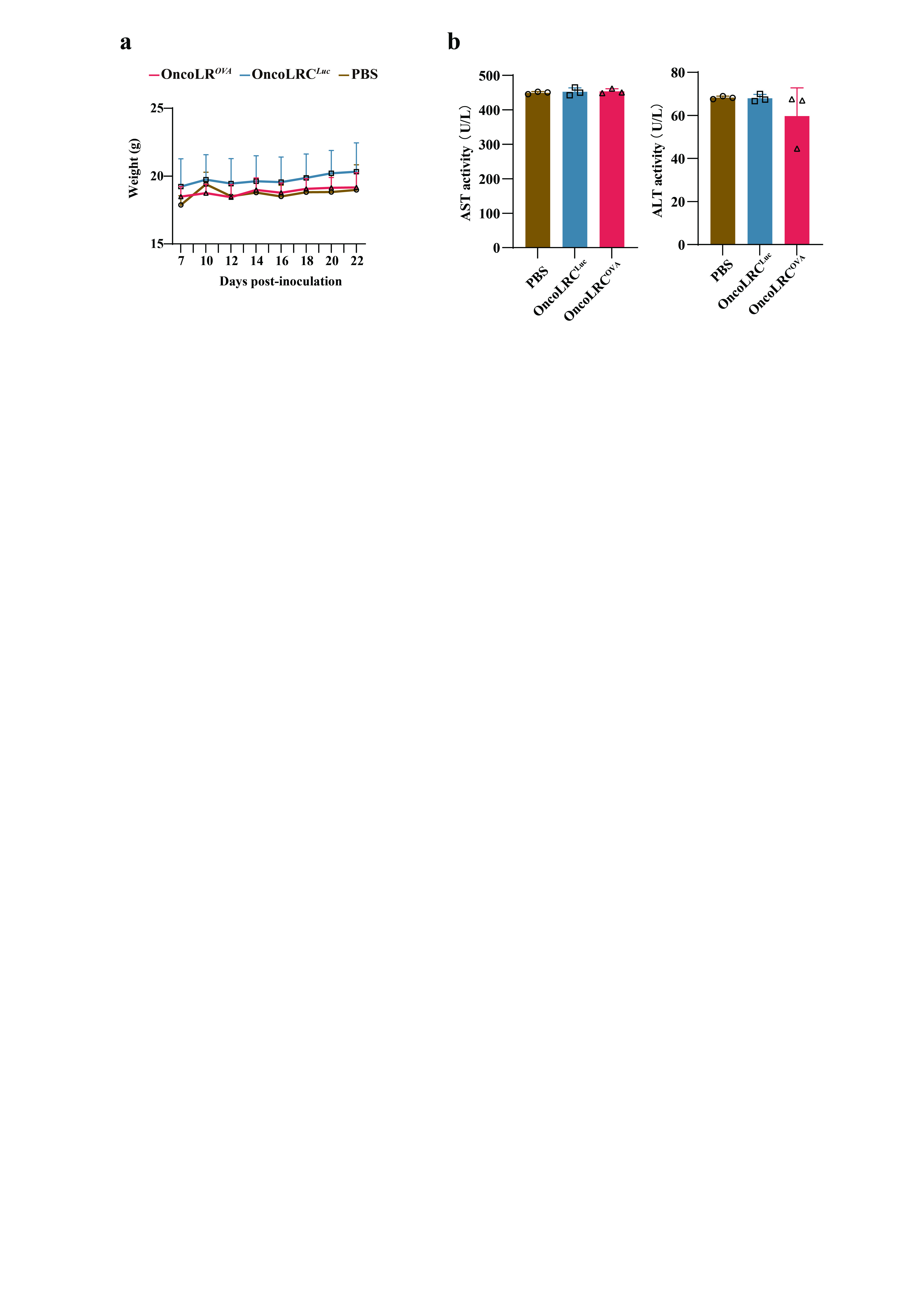


**Supplementary Figure 13.** (a) Body weight changes of mice in different treatment groups over time (n = 10). (b) Serum AST and ALT levels in subcutaneous B16F10-OVA tumor-bearing mice treated with different formulas. Serum samples were collected from different groups on day 37 (n = 3). Data are presented as mean ± SD.


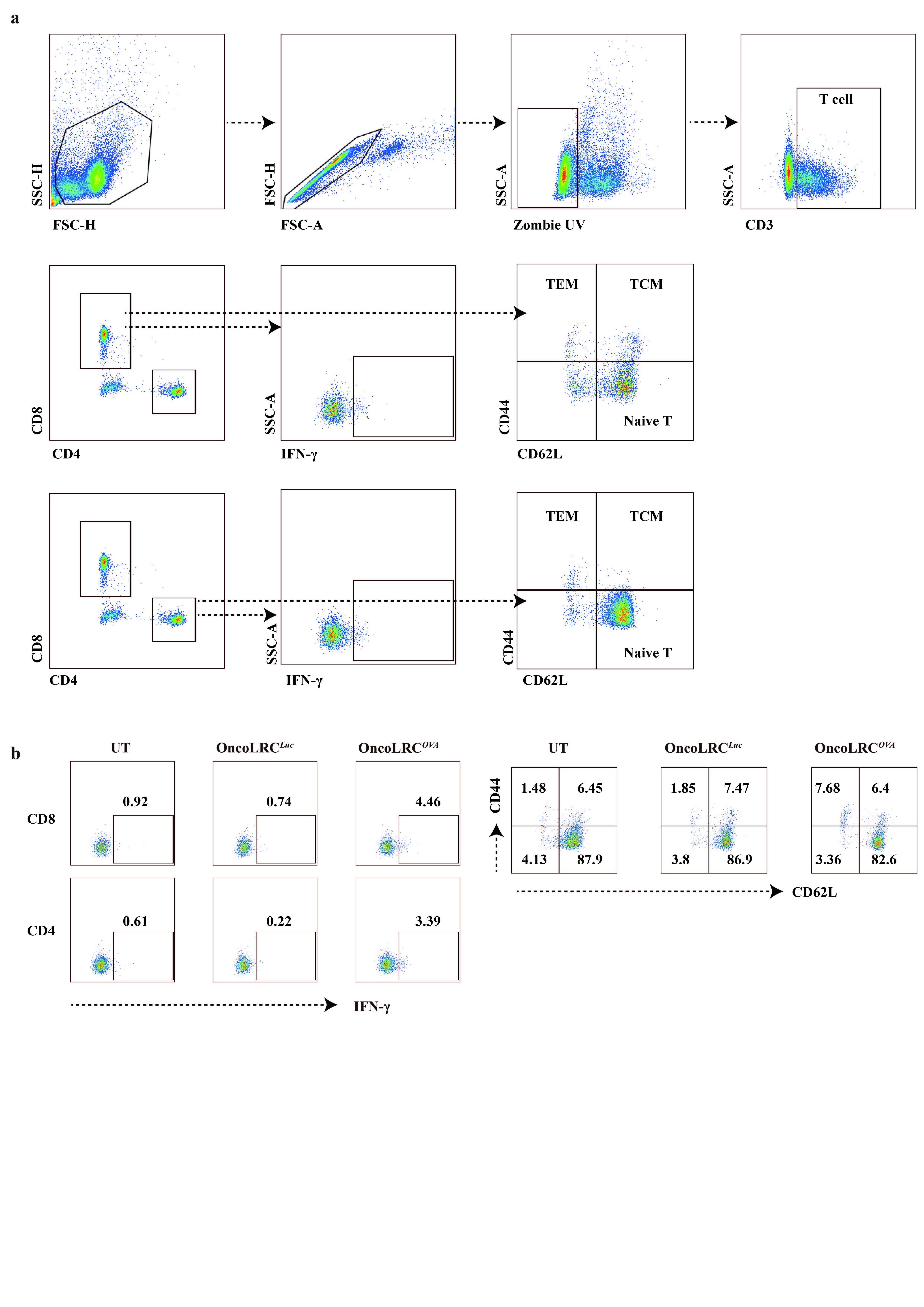


**Supplementary Figure 14.** (a) Gating strategy used for flow cytometry analyses of immune cells in the peripheral blood of B16F10-OVA tumor model after OncoLRC*^OVA^* treatment. (b) Representative FACS plots showing percentage changes of CD8^+^ cells, CD8^+^ IFNγ^+^ T cells, and memory T cells in the peripheral blood of mice from different treatment groups.


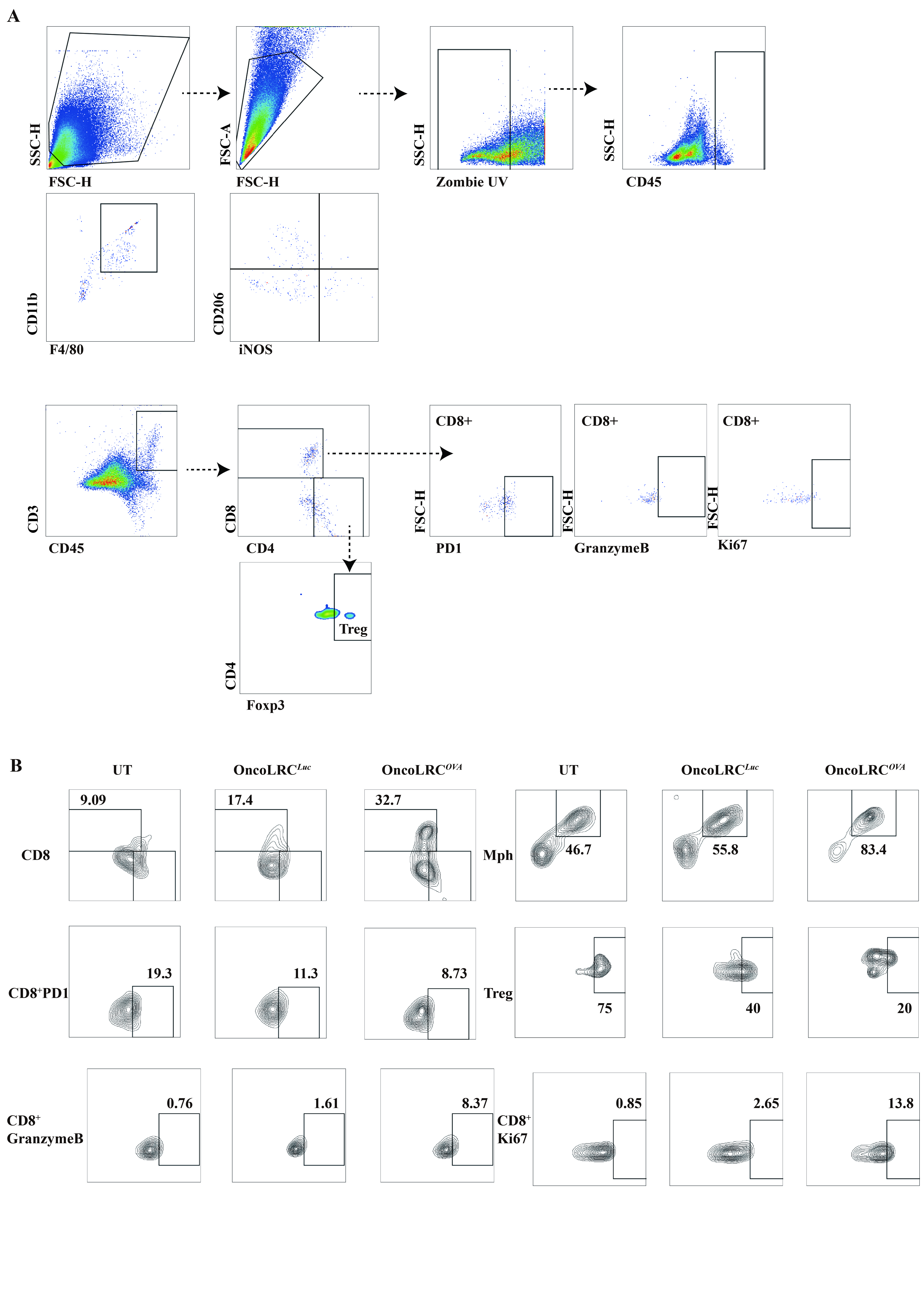


**Supplementary Figure 15.** (a) Gating strategy used for flow cytometry analyses of immune cells in B16F10-OVA TME after OncoLRC*^OVA^* treatment. (b) Representative FACS plots showing percentage changes of CD8^+^ cells, CD8^+^ PD1^+^ T cells, CD8^+^ Granzyme B^+^ T cells, CD8^+^ Ki67^+^ T cells, F4/80^+^ CD11b^+^ cells and FOXP3^+^ cells in the TME of mice from different treatment groups.


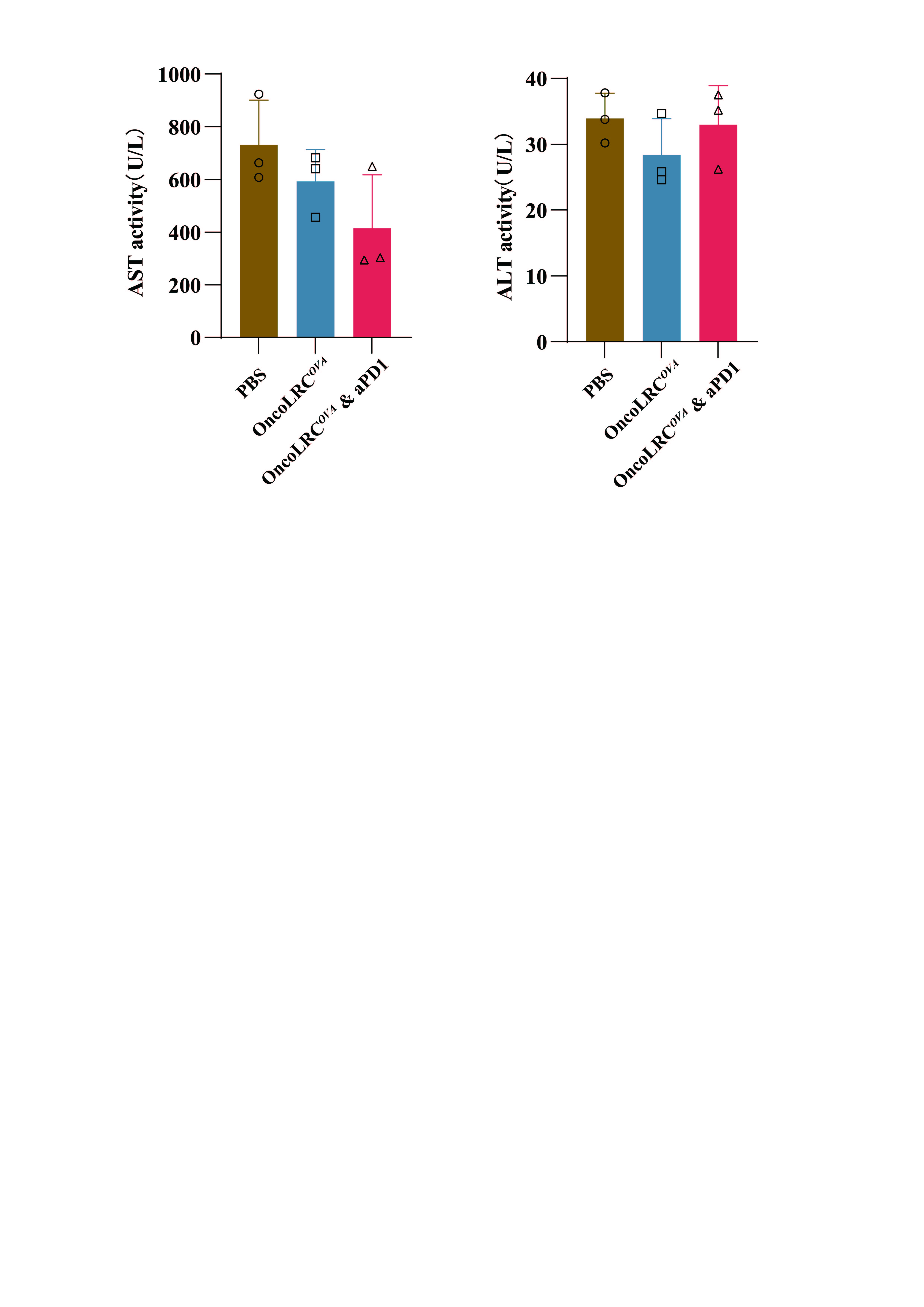


**Supplementary Figure 16.** Serum AST and ALT levels in subcutaneous B16F10-OVA tumor-bearing mice treated with different formulas. Serum samples were collected from different groups on day 37 (n = 3). Data are presented as mean ± SD.


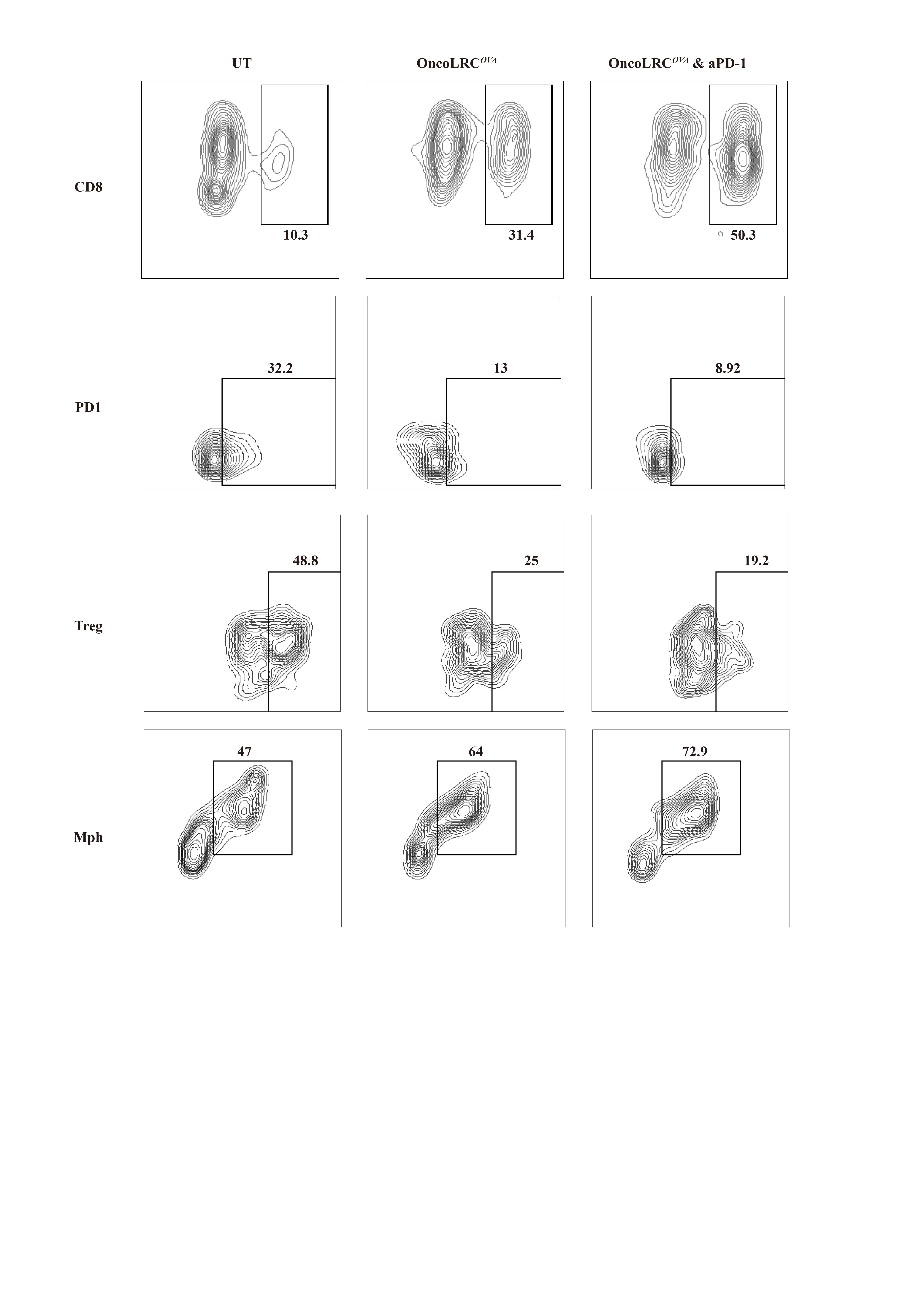


**Supplementary Figure 17.** Representative FACS plots showing percentage changes of CD8^+^ cells, CD8^+^ PD1^+^ T cells, F4/80^+^ CD11b^+^ cells and FOXP3^+^ cells in the B16F10-OVA TME of mice from different treatment groups.

**Supplementary Table 1.** Physicochemical properties of LNPs

| ID | Ratio | | | | Size*^a^* (nm) | PDI*^a^* | Zeta*^b^*  (mV) | p*K*a | EE (%) |
| --- | --- | --- | --- | --- | --- | --- | --- | --- | --- |
|  | Lipid | Chol | DSPC | DMG-PEG |  |  |  |  |  |
| Four components H2T7 LNP | 50.00 | 38.50 | 10.00 | 1.50 | 178 | 0.12 | 6.0 | 7.8 | 91.8 |
| OncoLRC | 100 | 0 | 0 | 0 | 158.2 | 0.11 | -51.0 | 7.1 | 84.8 |

*^a^*Determined by DLS in 150 mM NaCl at 25℃. *^b^*Measured by electrophoresis at 25℃.

**Supplementary Table 2.** Physicochemical properties of LPX

| ID | Ratio | | | | | | | Size*^a^* (nm) | PDI*^a^* | Zeta*^b^*  (mV) | p*K*a | EE (%) |
| --- | --- | --- | --- | --- | --- | --- | --- | --- | --- | --- | --- | --- |
|  | DOTMA | |  | | DOPE | |  |  |  |  |  |  |
| LPX | 50 |  | | 50 | |  | | 231.8 | 0.16 | -54.9 | 7.8 | 43.1 |

*^a^*Determined by DLS in 150 mM NaCl at 25℃. *^b^*Measured by electrophoresis at 25℃.

**Supplementary Table 3.** Antibodies used in this study

| Antibody | Source | Cat# |
| --- | --- | --- |
| CD45 Monoclonal Antibody (I3/2.3), APC-Cy™7 | BD Biosciences | 557659 |
| CD3 Monoclonal Antibody (17A2), PerCP-Cy™5.5 | BD Biosciences | 560527 |
| CD4 Monoclonal Antibody (RM4-5), Alexa Fluor 700 | Biolegend | 100536 |
| CD8a Monoclonal Antibody (53-6.7), BV605 | BD Biosciences | 563152 |
| NK1.1 Monoclonal Antibody (PK136), APC | Thermo Fisher Scientific | 17-5941-82 |
| CD11b Monoclonal Antibody (M1/70), BV650 | BD Biosciences | 563402 |
| CD11c Monoclonal Antibody (HL3), BV605 | BD Biosciences | 563057 |
| F4/80 Monoclonal Antibody (T45-2342), BV421 | BD Biosciences | 565411 |
| F4/80 Monoclonal Antibody (BM8), FITC | Thermo Fisher Scientific | 11-4801-82 |
| CD19 Monoclonal Antibody (6D5), APC/Fire™810 | Biolegend | 115577 |
| CD62L Monoclonal Antibody (W18021D), FITC | Biolegend | 161212 |
| CD44 Monoclonal Antibody (IM7), BV650 | Biolegend | 103049 |
| IFN-γ Monoclonal Antibody (XMG1.2), PE | BD Biosciences | 554412 |
| CD40 Monoclonal Antibody (HM40-3), FITC | Thermo Fisher Scientific | 11-0402-81 |
| CD86 Monoclonal Antibody (PO3), PE/Cyanine7 | Biolegend | 105116 |
| CD317 Monoclonal Antibody (927), APC | Biolegend | 127016 |
| iNOS Monoclonal Antibody (W16030C), APC | Biolegend | 696808 |
| CD206 Monoclonal Antibody (Y17-505), PE | BD Biosciences | 568273 |
| Foxp3 Monoclonal Antibody (150D/E4), PE | Thermo Fisher Scientific | 12-4774-42 |
| CD279 (PD-1) Monoclonal Antibody (RMP1-30), PE/Dazzle 594 | Biolegend | 109116 |
| Ki67 Monoclonal Antibody (16A8), FITC | Biolegend | 652409 |
| Granzyme B Monoclonal Antibody (GB11), FITC | BD Biosciences | 561998 |
| Zombie UV™ Fixable Viability Kit | Biolegend | 423108 |
| Perforin Monoclonal Antibody (S16009A), APC | Biolegend | 154303 |
| CD31 Monoclonal Antibody (390), PE-Cy™7 | BD Biosciences | 561410 |
